# *Fgfr3* mutation disrupts chondrogenesis and bone ossification in zebrafish model mimicking CATSHL syndrome partially via enhanced Wnt/β-catenin signaling

**DOI:** 10.7150/thno.45286

**Published:** 2020-05-30

**Authors:** Xianding Sun, Ruobin Zhang, Hangang Chen, Xiaolan Du, Shuai Chen, Junlan Huang, Mi Liu, Meng Xu, Fengtao Luo, Min Jin, Nan Su, Huabing Qi, Jing Yang, Qiaoyan Tan, Dali Zhang, Zhenhong Ni, Sen Liang, Bin Zhang, Di Chen, Xin Zhang, Lingfei Luo, Lin Chen, Yangli Xie

**Affiliations:** 1 Department of Wound Repair and Rehabilitation Medicine, State Key Laboratory of Trauma, Burns and Combined Injury, Daping Hospital, Army Medical University, Chongqing 400042, China.; 2 Research Center for Human Tissues and Organs Degeneration, Shenzhen Institutes of Advanced Technology, Chinese Academy of Sciences, Shenzhen 518055, China.; 3 Departments of Ophthalmology, Pathology and Cell Biology, Columbia University, New York, NY 10032, USA.; 4 Key Laboratory of Freshwater Fish Reproduction and Development, Ministry of Education, Laboratory of Molecular Developmental Biology, School of Life Sciences, Southwest University, Beibei, Chongqing 400715, China.

**Keywords:** FGFR3, CATSHL syndrome, Zebrafish, Skeletal development, Wnt/β-catenin

## Abstract

CATSHL syndrome, characterized by camptodactyly, tall stature and hearing loss, is caused by loss-of-function mutations of fibroblast growth factor receptors 3 (FGFR3) gene. Most manifestations of patients with CATSHL syndrome start to develop in the embryonic stage, such as skeletal overgrowth, craniofacial abnormalities, however, the pathogenesis of these phenotypes especially the early maldevelopment remains incompletely understood. Furthermore, there are no effective therapeutic targets for this skeleton dysplasia.

**Methods:** We generated *fgfr3* knockout zebrafish by CRISPR/Cas9 technology to study the developmental mechanisms and therapeutic targets of CATSHL syndrome. Several zebrafish transgenic lines labeling osteoblasts and chondrocytes, and live Alizarin red staining were used to analyze the dynamical skeleton development in *fgfr3* mutants. Western blotting, whole mount in situ hybridization, Edu labeling based cell proliferation assay and Wnt/β-catenin signaling antagonist were used to explore the potential mechanisms and therapeutic targets.

**Results:** We found that *fgfr3* mutant zebrafish, staring from early development stage, showed craniofacial bone malformation with microcephaly and delayed closure of cranial sutures, chondroma-like lesion and abnormal development of auditory sensory organs, partially resembling the clinical manifestations of patients with CATSHL syndrome. Further studies showed that *fgfr3* regulates the patterning and shaping of pharyngeal arches and the timely ossification of craniofacial skeleton. The abnormal development of pharyngeal arch cartilage is related to the augmented hypertrophy and disordered arrangement of chondrocytes, while decreased proliferation, differentiation and mineralization of osteoblasts may be involved in the delayed maturation of skull bones. Furthermore, we revealed that deficiency of *fgfr3* leads to enhanced IHH signaling and up-regulated canonical Wnt/β-catenin signaling, and pharmacological inhibition of Wnt/β-catenin could partially alleviate the phenotypes of *fgfr3* mutants.

**Conclusions:** Our study further reveals some novel phenotypes and underlying developmental mechanism of CATSHL syndrome, which deepens our understanding of the pathogenesis of CATSHL and the role of *fgfr3* in skeleton development. Our findings provide evidence that modulation of Wnt/β-catenin activity could be a potential therapy for CATSHL syndrome and related skeleton diseases.

## Introduction

CATSHL (camptodactyly, tall stature, and hearing loss) syndrome (OMIM 610474) is a genetic disorder first named and described by Toydemir et al. in 1996 1. Toydemir et al. evaluated a multigenerational family with 27 living affected members, all of whom possessed an autosomal dominant syndrome of camptodactyly, tall stature, and hearing loss 1. Then, Makrythanasis et al. reported an autosomal recessive case and Escobar et al. reported an autosomal dominant family with CATSHL Syndrome 2,3. Other clinical features of CATSHL syndrome include microcephaly, wormian skull bones, a high palate, pectus excavatum, scoliosis/kyphoscoliosis, tall vertebral bodies with irregular borders, broad femoral metaphysis, osteochondromas of the femur, tibia, or phalanx, and intellectual disabilities 1-3. Hearing loss in CATSHL syndrome appears to be sensorineural, congenital, and progressive in affected individuals 1.

CATSHL syndrome is caused by mutations in the fibroblast growth factor receptors 3 (*FGFR3*) gene on chromosome 4p16 1-3. In humans, FGFR3 is one of the four membrane-spanning receptor tyrosine kinases that serve as high affinity receptors for multiple fibroblast growth factors 4. It plays an essential role in the development of a wide range of tissues, especially the skeleton 5. Patients with activating FGFR3 mutations exhibit skeletal dysplasia characterized by short-limbed dwarfism including achondroplasia (ACH), hypochondroplasia, thanatophoric dysplasia I/II, Muenke syndrome, Crouzon syndrome with acanthosis nigricans, severe ACH with developmental delay and acanthosis nigricans (SADDAN) and lacrimo-auriculo-dental-digital (LADD) syndrome 4,6-8. Some patients with Muenke syndrome or Crouzon syndrome with acanthosis nigricans have premature closure of cranial sutures. Activated FGFR3 results in disordered endochondral bone growth and skeletal dysplasia through impaired proliferation and differentiation of growth plate chondrocytes 9,10. Similar to the phenotype of patients with CATSHL syndrome, *Fgfr3* deficient mice also have skeletal overgrowth due to enhanced proliferation of growth plate chondrocytes, and sensorineural deafness 11,12.

Physiological and pathological development of skeleton are gradually finished, starting from early embryonic stage. Although there are well designed studies about the skeleton phenotypes and underlying mechanisms of CATSHL patients or mice with FGFR3 deficiency, majority of them studied the skeleton phenotypes at perinatal or postnatal stages. The reason for few studies about the early skeleton phenotypes of *Fgfr3* mutants is that it is very difficult to analyze the early phenotype development process dynamically in current animal models such as mice. Furthermore, the *Fgfr3* deficient mice have no apparent phenotype in craniofacial skeleton 12,13, while CATSHL syndrome patients exhibit craniofacial skeleton phenotypes such as microcephaly, high palate and wormian bones 1-3. The reason for the discrepant in the craniofacial phenotypes between mouse model and patients remains to be studied. In addition, the molecular mechanism of the skeleton phenotypes especially the early maldevelopment of CATSHL syndrome is not well understood and therefore there are no effective therapies to alleviate the skeletal phenotypes.

Zebrafish (*Danio rerio*) has become an immensely useful and popular model for genetic and developmental studies. Due to its short developing time, external development, transparency and strong breeding ability, zebrafish are suitable for various genetic manipulation, living imaging observation and drug screens 14-17. Especially, the superior imaging technology available in zebrafish has provided unprecedented insights into the dynamics of skeletal development 18,19. In addition, the craniofacial bones of zebrafish are formed through both endochondral ossification and intramembranous ossification like humans. Especially the ceratohyal bone in zebrafish is composed of epiphysis and epiphysial growth plates and formed through endochondral ossification similar to that of mammalian 20. Previous studies have shown that mutation of *rmrp* and *nans* genes, which cause chondrodysplasia in humans, can also lead to maldevelopment of craniofacial bone such as ceratohyal cartilage in zebrafish 19,21. Furthermore, the fundamental signaling pathways and cellular events that sculpt the nascent craniofacial skeleton in the embryo have been proven to be highly conserved from fish to human 22,23. Therefore, zebrafish is a suitable model for studying the role and mechanism of *FGFs/FGFRs* in long bone and craniofacial skeleton development.

The roles of Fgfrs in zebrafish have been examined using morpholinos and dominant-negative approaches. Fgfr1a, Fgfr2 and Fgfrl1 were found to control the cranial cartilage development. Fgfr3c is required for the early embryogenesis and the anterior-posterior patterning of zebrafish 24-26. However, the function of Fgfr3 in zebrafish development especially the skeleton development has not been illuminated. The expression pattern of *fgfr3* in zebrafish has been found to be similar to those in higher vertebrates. Fgfr3 is expressed in chondrocytes of the head cartilages, osteoblasts, ventricular zone of the brain, undifferentiated mesenchymal cells of the skin, and eye lens epithelia 27.

In this study, we generated *fgfr3* knockout zebrafish to study the function of *fgfr3* in zebrafish skeleton development and gain novel insight into the mechanisms underlying the maldevelopment of skeleton, especially the early skeleton development of CATSHL syndrome. We found that *fgfr3* mutants, staring from 10 days post fertilization (dpf), gradually showed craniofacial bone malformation with smaller cranial skull bones and delayed closure of cranial sutures, as well as dysregulated development of pharyngeal arch cartilage with abnormal hypertrophy and disordered arrangement of chondrocytes. Further studies showed that *fgfr3* regulates the proliferation and differentiation of chondrocytes, as well as the osteogenic differentiation and mineralization of zebrafish craniofacial bone, and up-regulated IHH and Wnt/β-catenin pathway is involved in the maldevelopment of skeleton especially the cartilage in mutants.

## Materials and Methods

### Zebrafish strains and generation of transgenic lines

Zebrafish (*Danio rerio*) of the AB genetic background were used. The *Tg(col2a1a:EGFP)* was described before 28. The *Tg(osterix:EGFP)* is a gift from Chung-Der Hsiao (Chung Yuan Christian University, Chung-Li, Taiwan). All zebrafish were housed in semi-closed recirculation housing systems (ESEN, Beijing, China) and were kept at a constant temperature (27-28°C) on a 14:10 hour light: dark photoperiod. All zebrafish lines were raised and maintained according to standard protocols as previously described 29. All *in vivo* experiments and protocols were approved by Institutional Animal Care and Use Committee of the Research Institute of Surgery, Daping Hospital IACUC protocol SYXK-(Army) 2017-0057.

### Multiple sequence alignment of FGFR3

ClustalW software was used for multiple sequence alignment. Amino acids of GenBank Accession numbers: human FGFR3: NM_000142, mouse FGFR3: NM_001163215 and zebrafish Fgfr3: NM_131606 were used for alignment. Consensus elements were highlighted with BOXSHADE.

### *In situ* hybridizations

The template for *fgfr3* antisense probe synthesis was amplified with the following primer, forward primer: 5'-GGTGACCTTGGAAAGGATTACTG-3', reverse primer: 5'-CCTGCCTCGTCCTCATCTTCATC-3'. Primers for other probes are available from the corresponding authors upon request. PCR products were cloned into the pGEMT-easy vector (Promega). Digoxigenin-labelled probes were generated by *in vitro* transcription (DIG RNA Labeling Kit, Roche) and *in situ* hybridization was carried out as previously described 30. The WISH images were captured using a SteREO Discovery 20 microscope (Carl Zeiss).

### Generation of *fgfr3* mutants using CRISPR/Cas9 system

*Fgfr3* mutants were generated by targeting the 11th exon of *fgfr3* with CRISPR/Cas9 technology. The process was performed as previously described 31. The *fgfr3* target sequence was 5'-GGGAGAGGGCTGCTTTGGGC-3', and the target region was amplified by PCR using the following primers: forward primer: 5'-GCCTAACGTGTCTGAACTTGAAC-3', reverse primer: 5'-GATGGCATTTGAATATACACTCAC-3'. Founder fish (F0) carrying deletion mutations and corresponding F1 embryos were raised up. F1 fish with heterozygote* fgfr3* mutation were crossed to identify mutants with phenotypes.

### X -ray and micro-computed tomography

3-month adult zebrafish (equal to 26.0 mm SL (standard length)) were harvested, stored in 75% ethanol at 4°C, and were subjected to high-resolution X-rays examination using Faxitron MX20. The zebrafish were scanned with micro-CT (viva CT-40, Scanco Medical AG, Switzerland). Image acquisition was performed with the condition of 45 kV and 177 μA in high-resolution scans (10.5µm voxel resolution). Two-dimensional images were used to generate three-dimensional reconstructions. Every measurement used the same filtering and segmentation values. The images were analyzed by micro-CT Evolution Program V6.5 software.

### Edu cell proliferation assay

The Edu cell proliferation assay was applied for S-phase labeling according to the manufacturer's instructions (Click-iT Edu Imaging Kits, Invitrogen, C10340). Wild-type (WT) and *fgfr3* mutants were injected with 10 nL 0.2 mM Edu (Life Technology, E10187) at 1 month (SL 10.0 mm) and incubated at 28.5°C incubator for 2 hours. After being fixed with 4% formaldehyde at 4°C overnight, the samples were subjected to frozen section and EdU assay.

### Western blot analysis

Zebrafish head skeleton (*n* = 20) at 20 dpf (SL 7.5 mm) and 40 dpf (SL 13.0 mm) were dissected and lysed using RIPA lysis buffer containing protease inhibitors (Roche). Equal amount of protein samples was resolved on a 12% SDS-PAGE gel and transferred onto a PVDF membrane (Millipore). Then samples were probed with primary antibody specific for non-phospho (active) β-catenin (1:1000; Cell Signaling Technology, 8814S), phospho-β-catenin (1:1000; Cell Signaling Technology, 9561S), IHH (1:1000; Abcam, ab52919), followed by secondary antibodies. The signal was detected using the chemiluminescent signal (Pierce, NCI4106) according to the manufacturer's instructions. Western blot analysis was performed for three times. Quantitative analysis for western blotting was conducted using ImageJ software.

### Skeletal analysis by whole skeleton staining, histology and imaging

Zebrafish *in vivo* skeletal staining was incubated with 0.05% Alizarin red (Sigma, A5533) for 1 hour and then washed with system water three times. Alizarin red and Alcian blue whole skeleton staining was performed as described 12. For histological analysis, samples were fixed in 4% paraformaldehyde, decalcified in 15% EDTA and embedded in paraffin as described 32. Sections (5-μm thick) were stained with Safranin O/Fast Green and hematoxylin and eosin (H & E). Picric-sirius red staining was used to detect collagen fiber density and organization as described 33. Zebrafish embryos were imaged with a SteREO Discovery 20 microscope (Carl Zeiss) or LSM880NLO confocal microscope with a 20× water immersion objective (Carl Zeiss). The ceratohyal length measurements, the relative area of osterix labeled osteoblasts measurements and the mineralization intensity measurement were conducted using Image J.

### Drug treatment

XAV939 was purchased from Selleck Chemicals and was dissolved with DMSO to get a stock solution of 10 mM. The drug treatment was performed for three times. In each experiment, the self-crossing offspring of *fgfr3* heterozygous mutants were randomly assigned to be treated with 2.5 µM XAV939 or 0.1% DMSO control from 10 dpf (SL 5.0 mm) to 20 dpf (SL 7.5 mm) to investigate the effect of Wnt signaling antagonist on the development of pharyngeal arches cartilage. In each experiment, more than 60 WT and 20 *fgfr3* mutants were treated with DMSO or XAV939, respectively.

### Statistical analysis

All numeric data are presented as mean ± SD. Error bars indicate SD. Differences between two groups were evaluated using Unpaired Student's t test, and ANOVA was used for comparisons of multiple groups. When significant levels (P<0.05) were achieved, Tukey's Post Hoc test was performed. All statistical analyses were performed using GraphPad PRISM 7.0 software, and P-values were considered significant at * p < 0.05, ** p < 0.01, *** p < 0.001.

## Results

### Zebrafish *fgfr3* is highly conserved across multiple species

**Figure 1:**
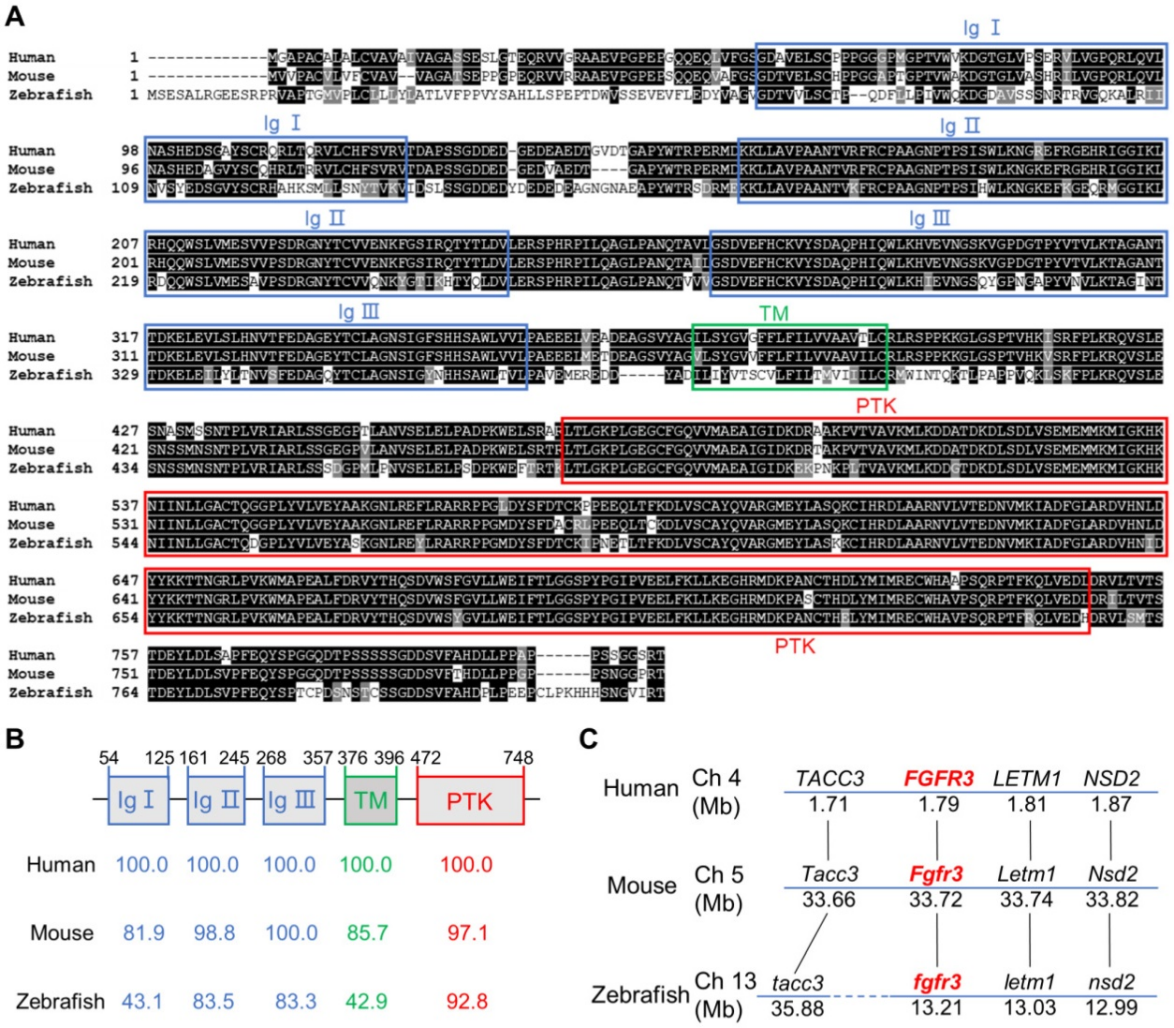
** Zebrafish *fgfr3* is highly conserved across multiple species. (A)** Multiple alignment of amino acids of FGFR3 for human (806 aa), mouse (800 aa) and zebrafish (819 aa). Identical amino acids are shaded. The three Ig-like domains (Ig I-III), transmembrane domain (TM), and tyrosine-protein kinase domain (PTK) are marked with blue, green and red boxes, respectively. **(B)** The identity of multiple domains of each FGFR3 protein for human, mouse and zebrafish, as referred to human FGFR3. **(C)** Conserved synteny analysis for *FGFR3* gene in zebrafish, human and mouse. Numbers next to the gene names represent megabase pair (Mbp) of gene location on the chromosome. Chromosome segments are represented with blue lines and dashed blue lines represent discontinuous segments. Orthologs are connected with black lines.

To determine the sequence conservation of zebrafish* fgfr3*, we did multiple sequence alignments for coding sequence and amino acids of FGFR3 in human, mouse and zebrafish. We found that the amino acid identity of *FGFR3* was 76.9% between zebrafish and human, and 74.6% between zebrafish and mouse (Figure 1A). The homology of multiple domains of FGFR3 is variable with the amino acid identity of tyrosine-protein kinase catalytic domain as high as 92.8% between zebrafish and human, while that of three immunoglobulin (Ig)-like domain (Ig I-III) was 43.1%, 83.5%, 83.3% respectively. In contrast, the transmembrane domain shared only 42.9% identity (Figure 1A, B). We further compared the conservation of coding sequence of *fgfr3* and found a 59.41% sequence identity between zebrafish and human. We also performed a synteny analysis for *fgfr3* gene in zebrafish, human and mouse (Figure 1C). It showed that the surrounding genes (*LETM1*, *NSD2*, *TACC3*) of *FGFR3* in human and mouse have the similar positions as the surrounding genes (*letm1*, *nsd2*, *tacc3*) linked to *fgfr3* in zebrafish. These data suggest that zebrafish* fgfr3* gene is syntenic to human *FGFR3* and mouse* Fgfr3* gene. Together, these data suggest that zebrafish *fgfr3* is highly conserved especially in important catalytic domain and may play a conservative role across these species.

### Expression pattern of *fgfr3* during early development of zebrafish

**Figure 2:**
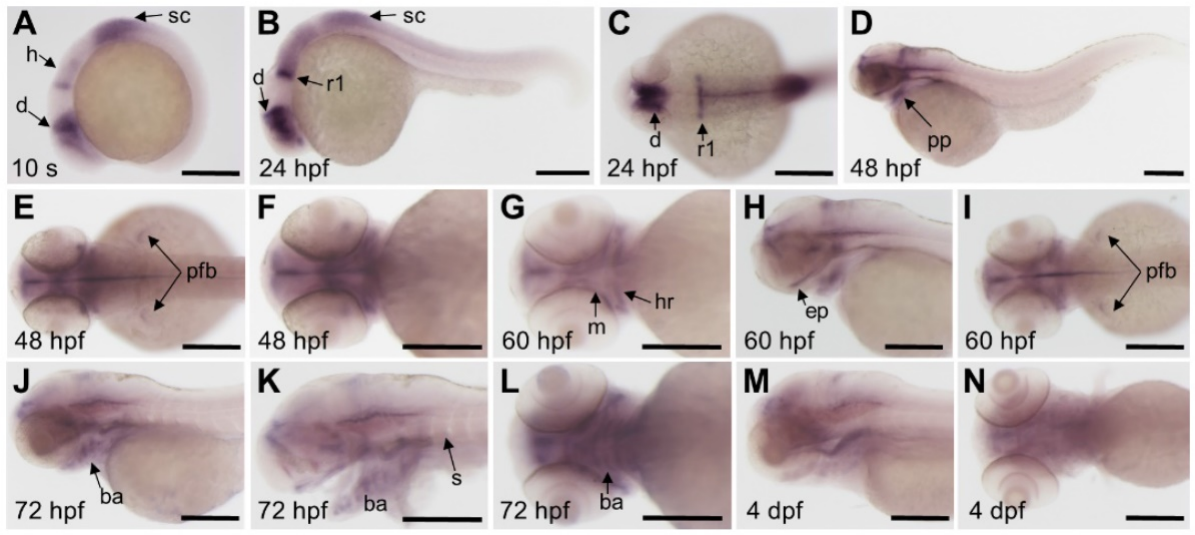
** Expression patterns of *fgfr3* detected by whole mount *in situ* hybridization in wild-type zebrafish. (A)** Lateral view of 10-somite stage; **(B-C)** show 24 hpf embryo in lateral view (B) and head region in dorsal view (C). **(D-F)** show 48 hpf embryo in lateral view (D) and head region in dorsal view (E) and in ventral view (F). **(G-I)** show head region of 60 hpf embryo in ventral view (G), in lateral view (H) and in dorsal view (I). **(J-L)** show head region of 72 hpf embryo in lateral view with yolk (J) and in lateral view without yolk (K), 72 hpf embryo in ventral view (L); **(M-N)** show head region of 4 dpf embryo in lateral view (M) and in ventral view (N). *n* = 30 embryos for A-N. *Abbreviations*: ba, branchial arch; d, diencephalon; ep, ethmoid plate; h, hindbrain; ha, hyoid arch; m, mandibular arch; pfb, pectoral fin bud; pp, pharyngeal pouches; r1, rhombomere 1; s, somites; sc, spinal cord. Scale bars: 200 µm in A-N.

We detected the expression pattern of *fgfr3* gene by whole mount *in situ* hybridization during early development stage. Results showed that* fgfr3* transcripts were first detected weakly at tailbud stage in the prospective diencephalon and the anterior hindbrain (Figure 2A). From segmentation stage to 24 hours post fertilization (hpf), *fgfr3* showed expression in diencephalon, anterior hindbrain, anterior spinal cord, especially was intensely expressed in the posterior rhombomere 1 (Figure 2B, C). *Fgfr3* transcripts were detected in pharyngeal pouches and the pectoral fin bud at 48 hpf (Figure 2D-F), and then in mandibular and hyoid arches cartilage at 60 hpf. It was also detected extensively in ethmoid plate at 60 hpf (Figure 2G-I). By 72 hpf, *fgfr3* was expressed in chondrocytes of cartilage of branchial arch 1-5 (Figure 2J-L), and gradually diminished at 4 dpf (Figure 2M, N). Besides, *fgfr3* was weakly expressed in the somites. These observations suggest a potential role for *fgfr3* in the development of various tissues, including cartilage.

### *Fgfr3* deletion in zebrafish disrupts craniofacial skeleton development

**Figure 3:**
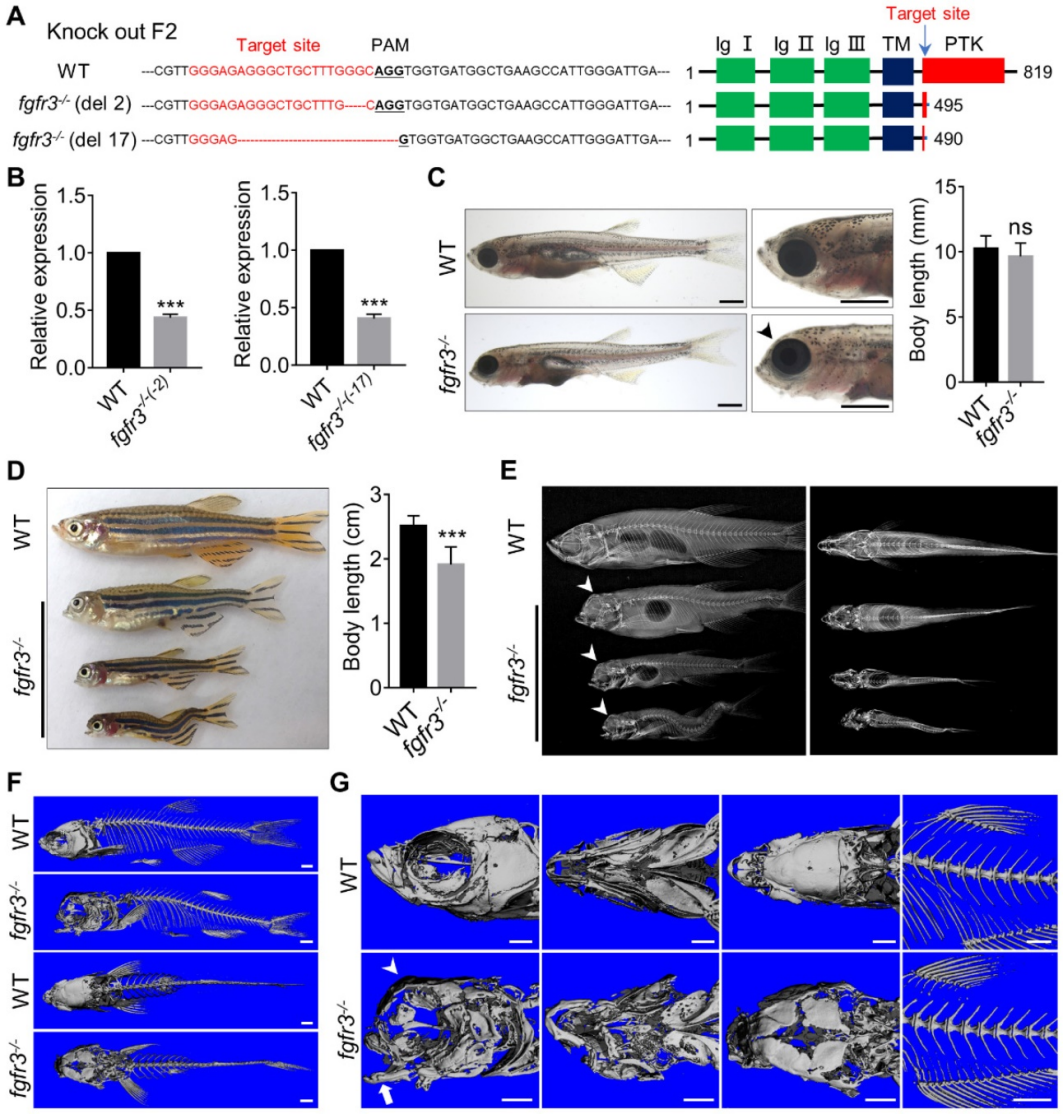
***Fgfr3* knockout in zebrafish disrupts craniofacial development. (A)** The WT and two *fgfr3* mutant lines in F2 generation generated by CRISPR/Cas9 technology. Left panel show the target sequence (red), PAM sequence (bold dashed line) and 2 bp or 17 bp deletion of the mutant lines. Right panel diagram domains of WT and predicted mutants of Fgfr3 protein. **(B)** RT-qPCR analysis of the expression level of *fgfr3* in 2 bp deletion mutant line and 17 bp deletion mutant line at 20 dpf (SL 7.5 mm). *n* = 3 independent experiments. ***p < 0.001. **(C, D)** Bright-field images showing the WT and *fgfr3* mutant at 1 month (SL 10.0 mm) (C) and the adult stage about 3 months (SL 26.0 mm) with different degrees of skull deformity (D). The quantification of body length for (C) and (D) is show in the right panel. *n* = 10, ***p < 0.001, no significance (ns). **(E)** X-rays of the corresponding zebrafish of (D) in lateral view (left) and dorsal view (right). **(F, G)** The micro-CT reconstruction images of WT and *fgfr3* mutant, the (F) are magnified in the (G). Note that the domed-shaped skull (arrowheads), drooped hyoid arch (arrow), malformed jaw bone and delayed closure of cranial sutures in *fgfr3* mutants. Scale bars: 1 mm in C, F-G.

In order to generate the zebrafish model of CATSHL and study the role of *fgfr3* in development, we used CRISPR/Cas9 genome editing approach to knockout *fgfr3* by targeting the tyrosine-protein kinase domain of Fgfr3 protein. We identified two stable homozygous mutant lines in F2 generation, which have 2-bp and 17-bp deletion, respectively (Figure 3A). Both two mutations caused a frameshift, predictably resulting in premature stop codon with a truncated Fgfr3 protein lacking the tyrosine kinase domain (Figure 3A). We performed real-time quantitative PCR (RT-qPCR) and found that the expression level of* fgfr3* was significantly decreased in both two *fgfr3* mutant lines (Figure 3B). We analyzed the gross phenotypes of *fgfr3* knockout zebrafish in F3 generation. Since we found the same phenotype in these two mutant lines, we chose the 17-bp deletion line as *fgfr3* mutant in our following studies. *Fgfr3* heterozygous mutants were normal as wild-type (WT) siblings. Homozygous mutant embryos showed no obvious morphological defects until at 1 month (SL 10.0 mm) when the jawbone and skull bone were gradually formed. The *fgfr3* mutants were readily identified by their mandibular deformity with hyoid arch drooping toward the ventral side and mild domed-shaped skulls at 1 month (SL 10.0 mm) (Figure 3C), which resemble the microcephaly and high palate in the skulls of CATSHL patients 3, but the body length of WT and *fgfr3* mutant had no significant change at 1 month (SL 10.0 mm). Some *fgfr3* mutants can't grow to adult stage due to severe cranial deformity, while mutants survived to adult stage had smaller body size than that of WT siblings (Figure 3D). From gross appearance and X-rays, we can find that *fgfr3* mutants exhibited varying degrees of mandibular deformity with remarkable domed-shaped skulls and microcephaly. Especially in *fgfr3* mutants with severe phenotypes, their mouth failed to close due to severe jaw deformity with resultant eating disorder (Figure 3D, E).

We performed high resolution micro-CT and three-dimensional skeletal reconstructions of adult zebrafish and found that the ossification of the craniofacial bone including mandibular bone and skull bone was severely decreased in *fgfr3* mutants (Figure 3F, G). The *fgfr3* mutants exhibited grossly deformed pharyngeal arches, domed skulls, microcephaly, midface hypoplasia, and delayed closure of cranial sutures (Figure 3 F, G). The spine in most mild and moderate *fgfr3* mutants was morphologically normal, but exhibited kyphosis and mild scoliosis in severe *fgfr3* mutants (Figure 3E). The vertebral body, neural arches and haemal arches had no significant abnormality in *fgfr3* mutants compared to the WT (Figure 3F, G).

Alizarin red and Alcian blue whole skeleton staining were performed to further examine the changes of skeleton in *fgfr3* mutants (Figure S1A, B). We found the consistent morphological changes of craniofacial skeleton as micro-CT revealed. We then dissected the craniofacial bone and interestingly found some small bone islands at the margin of the cranial bone in *fgfr3* mutants (Figure S1A), which resemble wormian bones in the skull of CATSHL patients 2. Cranial base will affect the shape of skulls 34; we found delayed synchondrosis closure and irregular morphology of the cranial base in *fgfr3* mutants (Figure S1A). Both the mandibular arch and hyoid arch had symmetrical and regular shape in WT, but they exhibited asymmetrical and irregular morphology in *fgfr3* mutants (Figure 3G and Figure S1A). The morphology of precaudal vertebrae and caudal vertebrae showed no marked abnormality in *fgfr3* mutants in contrast to WT. But the joint between the precaudal vertebrae and the ribs had abnormal morphology and more regions positively stained by Alcian blue. Besides, the caudal fin vertebrae were slightly bent with abnormal morphology (Figure S1B).

### *Fgfr3* regulates timely ossification of craniofacial skeleton

**Figure 4:**
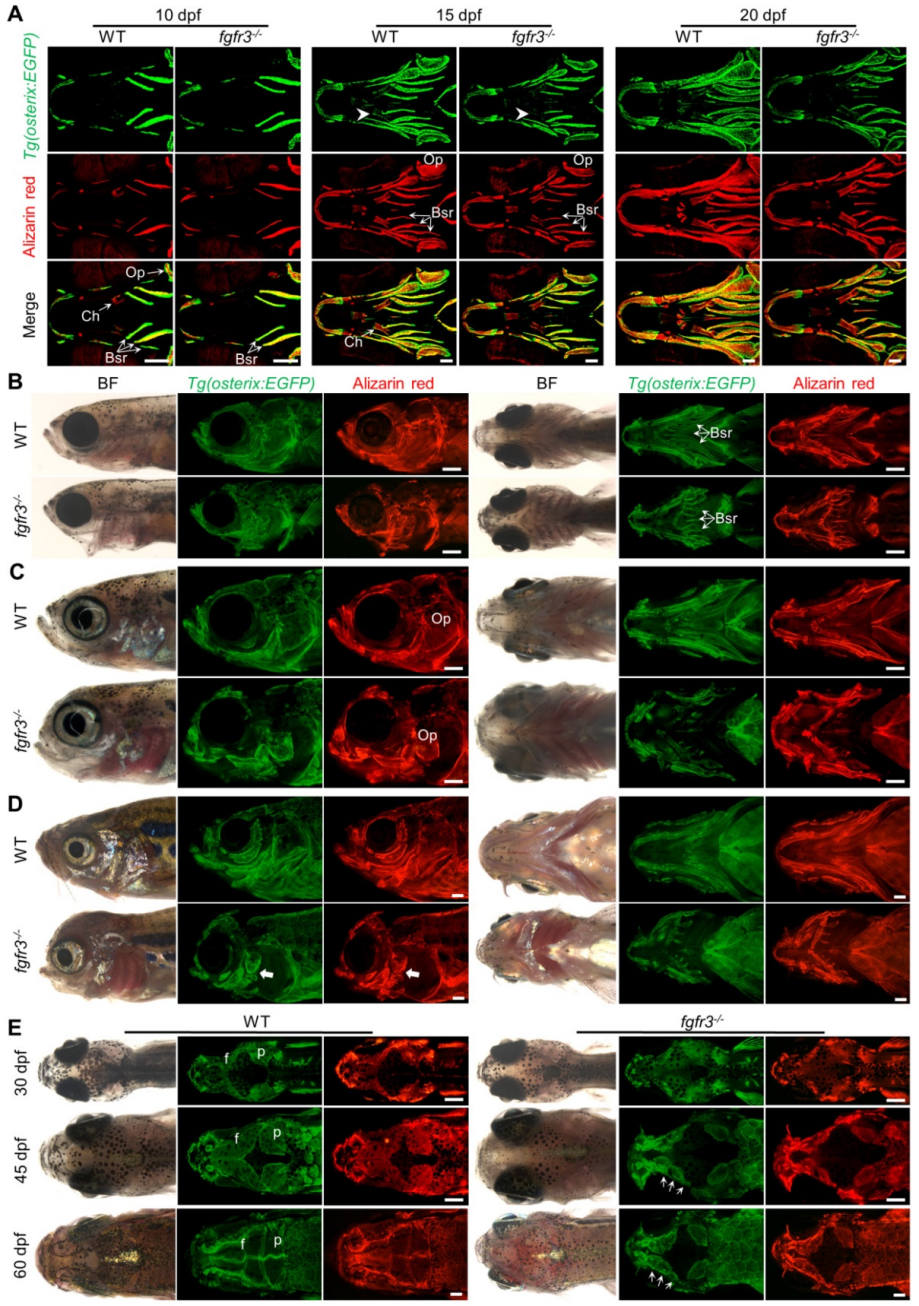
***Fgfr3* is required for the timely bone ossification. (A)** Confocal imaging of WT and* fgfr3* mutants in *Tg(osterix:EGFP)* background live stained with Alizarin red at 10 dpf (SL 5.0 mm) (left), 15 dpf (SL 6.0 mm) (middle) and 20 dpf (SL 7.5 mm) (right). White arrowheads indicate less osteoblasts at the mineralized bone collars of ceratohyal perichondrium. (B-D) Stereo fluorescence microscope imaging of WT and* fgfr3* mutants at 30 dpf (SL 10.0 mm) **(B)**, 45 dpf (SL 14.0 mm) **(C)** and 60 dpf (SL 18.0 mm) **(D)** in lateral view (left) and ventral view (right) after live stained with Alizarin red in *Tg(osterix:EGFP)*. White arrows indicate some small bone islands at the margin of the opercle bone. **(E)** Bright field (left), *Tg(osterix:EGFP)* images (middle) and Alizarin red staining images (right) showing the development of parietal (p) and frontal (f) bones of WT and* fgfr3* mutants in dorsal view at 30 dpf (SL 10.0 mm) (left), 45 dpf (SL 14.0 mm) (middle) and 60 dpf (SL 18.0 mm) (right). White arrows indicate several small separated bones in *fgfr3* mutants. *Abbreviations*: Bsr, branchiostegal rays; Ch, ceratohyal bone; Op, opercle. Scale bars: 100 µm in A, 400 µm in B-E.

It has been reported that both CATSHL patients and *Fgfr3* knockout mice have abnormal bone ossification 1,13. To study the effect of *fgfr3* mutation on bone formation in zebrafish at early embryonic development, we used live Alizarin red staining and transgenic line *Tg(osterix:EGFP)* that labels osteoblasts to examine the ossification in *fgfr3* mutants from 5 dpf (SL 4.0 mm) to 20 dpf (SL 7.5 mm). At 5 dpf (SL 4.0 mm), confocal microscopy revealed no significant difference in the bone ossification between WT and *fgfr3* mutants (Figure S2A, B). At 10 dpf (SL 5.0 mm), however, the *fgfr3* mutants showed delayed ossification of ceratohyal perichondrium and slightly delayed formation of branchiostegal rays (Figure 4A). Similar results were also observed using Alizarin red staining after fixation, which showed that the perichondral ossification of occipital arch in *fgfr3* mutants was delayed at 10 dpf (SL 5.0 mm) (Figure S3A). Whole mount *in situ* hybridization revealed that the expression of *col10a1*, which is related to osteoblast differentiation in zebrafish 35, was down-regulated in *fgfr3* mutants at 8 dpf (Figure S3B). We also found that the expressions of *col1a2*, *osteopontin* (*spp1*), *osteonectin* (*osn*), which were the later markers of osteoblast differentiation, were decreased in *fgfr3* mutants (Figure S3C). From 15 dpf (SL 6.0 mm) to 20 dpf (SL 7.5 mm), although the morphology of skulls still showed no gross change, confocal microscopy revealed that the ossification of intramembranous bone especially the opercle and branchiostegal rays was strongly inhibited (Figure 4A and Figure S4A). Besides, there were less osteoblasts at the mineralized bone collars of ceratohyal bone in *fgfr3* mutants (Figure 4A). These data indicate that the formation of both intramembranous bone and endochondral bone was delayed in *fgfr3* mutants in contrast to WT.

From 1 month (SL 10.0 mm), 1.5 month (SL 14.0 mm) to 2 months (SL 18.0 mm), both intramembranous bone and endochondral bone were gradually developed into a matured skeleton in WT skulls, however, the ossification of most intramembranous bones especially the opercle and branchiostegal rays were severely delayed in *fgfr3* mutants (Figure 4B-D and Figure S4B). In the margins of these immature intramembranous bones, there were small scattered bone islands that cannot develop into fully matured bones, indicating that the osteoblastic differentiation and mineralization were severely affected in *fgfr3* mutants. Besides, the symmetrical and regular shapes of mandibular arch and hyoid arch were gradually lost and became asymmetrical from 1 month (SL 10.0 mm) to 2 months (SL 18.0 mm) in *fgfr3* mutants (Figure 4B-D). Moreover, the gills were still exposed without being covered by the opercle and branchiostegal rays in mutants at 2 months (SL 18.0 mm) due to severe mineralization defect (Figure 4D).

As gain-of-function mutations of FGFR3 lead to craniosynostoses in both ACH patients and mouse model 7,36,37, Alizarin red staining and transgenic line *Tg(osterix:EGFP)* that labels osteoblasts were employed to image the cranium development *in vivo* dynamically from 1 month (SL 10.0 mm), 1.5 month (SL 14.0 mm) to 2 months (SL 18.0 mm). The cranial vault development is normally coordinated with that of brain. Parietal and frontal bones are formed through intramembranous ossification within a layer of mesenchyme positioned between the dermal mesenchyme and meninges surrounding the brain in zebrafish 38. In contrast to the WT, the intramembranous ossification of parietal and frontal bones was severely delayed in *fgfr3* mutants, leading to smaller bone that is unable to cover the brain at 2 months (SL 18.0 mm) (Figure 4E and Figure S4C). Both the frontal suture and coronal suture were not closed even at adult stage in *fgfr3* mutants. Besides, compared to the single whole bone in WT, the parietal and frontal bones in the mutants were consisted of several small and separately located bones that were unable to develop into fully matured single bone (Figure 3G, 4E, Figure S1A and Figure S4C), indicating that the osteoblastic differentiation and mineralization of cranial vault was severely blocked in *fgfr3* mutants.

### *Fgfr3* knockout inhibits scale formation

Zebrafish scales are part of the exoskeleton; their formation is needed for proper bone mineralization. Fgf signaling has been implicated in zebrafish scale development: *fgfr1a* and *fgf20a* mutations affect scale number and size in adult zebrafish 39-41. In addition to study the effect of *fgfr3* deficiency on craniofacial skeleton bone ossification, we also investigated the scale formation in *fgfr3* mutants. Using *Tg(osterix:EGFP)* transgenic line, we found that there were four rows of scales formed in WT at 35 dpf (SL 12.0 mm), but* fgfr3* mutants with similar body length only had one row of scales that just began to be formed (Figure S5A). At 50 dpf (SL 16.0 mm), the scales in WT exhibited shapes with regular smooth arc, but the mutant scales were smaller with irregular shapes. Besides, using live Alizarin red staining, we found that the mineralization of scales was reduced in *fgfr3* mutants (Figure S5B, C). These data indicate that *fgfr3* regulates the mineralization of scales, which is necessary for the scale formation.

### *Fgfr3* deficiency disrupts the patterning and shaping of pharyngeal arches

**Figure 5:**
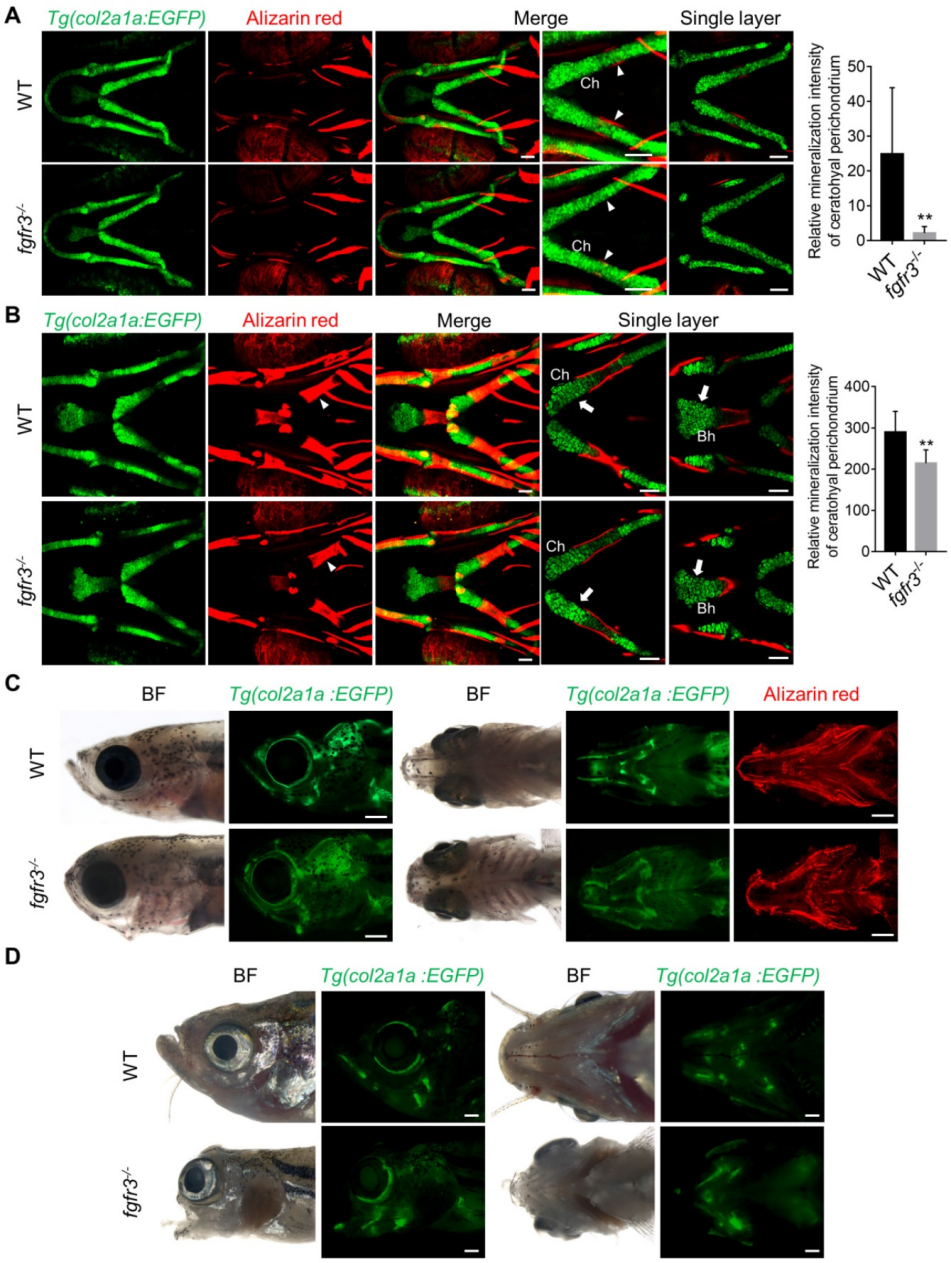
***Fgfr3* regulates the patterning and shaping of pharyngeal arche. (A-B)** Confocal imaging of WT and* fgfr3* mutants in *Tg(col2a1a:EGFP)* background live stained with Alizarin red at 10 dpf (SL 5.0 mm) (A) and 20 dpf (SL 7.5 mm) (B). From left to right are 3D view of* Tg(col2a1a:EGFP)* image, Alizarin red staining image, merged 3D view image and merged single layer image. The right panel are the quantification of relative mineralization intensity of ceratohyal perichondrium for WT and *fgfr3* mutants. White arrowheads indicate that perichondral ossification of ceratohyal cartilage was delayed in *fgfr3* mutants. White arrows indicate disarrangement of chondrocytes of ceratohyal cartilage and basihyal cartilage in *fgfr3* mutants in contrast to WT. *n* = 10, **p < 0.01. **(C)** Stereo fluorescence microscope imaging of WT and* fgfr3* mutants at 30 dpf (SL 10.0 mm) in lateral view (left) and ventral view (right) after live stained with Alizarin red in *Tg(col2a1a:EGFP)*. **(D)** Stereo fluorescence microscope imaging of WT and* fgfr3* mutants at about 3 months (SL 26.0 mm) in lateral view (left) and ventral view (right) in *Tg(col2a1a:EGFP)*. Ch: ceratohyal cartilage; Bh: basihyal cartilage. Scale bars: 50 µm in A and B, 400 µm in C and D.

It has been reported that both CATSHL syndrome patients and *Fgfr3* deficient mice exhibit skeletal overgrowth due to enhanced proliferation of growth plate chondrocytes 1-3, 11, 12. We used transgenic line* Tg(col2a1a:EGFP)* that labels chondrocytes to examine the early cartilage development in *fgfr3* mutants from 5 dpf (SL 4.0 mm) to 20 dpf (SL 7.5 mm). Confocal microscopy revealed that the morphology and arrangement of mandibular chondrocytes were similar to that in the WT at 5 dpf (SL 4.0 mm) (Figure S2C) and 10 dpf (SL 5.0 mm) (Figure 5A), but the perichondral ossification of ceratohyal cartilage was delayed in *fgfr3* mutants at 10 dpf (SL 5.0 mm) (Figure 5A). At 20 dpf (SL 7.5 mm), the gross features of pharyngeal arches still had no significant change, but the arrangement of ceratohyal and basihyal chondrocytes was gradually disordered in *fgfr3* mutants, with variable cellular sizes and polarity in contrast to the regular size and arrangement of chondrocytes in WT (Figure 5B). In addition, the perichondral ossification of ceratohyal cartilage was delayed severely (Figure 5B). At 30 dpf (SL 10.0 mm), the *fgfr3* mutants can be grossly distinguishable by their abnormally drooped hyoid arches (Figure 5C). Confocal microscopy revealed that the endochondral ossification of ceratohyal and palatoquadrate cartilage was delayed with disorganized chondrocyte arrangement especially in the ceratohyal cartilage of *fgfr3* mutants (Figure 7C, D). In addition, the morphology of mutant pharyngeal arches including the mandibular joint was abnormal with loss of symmetry (Figure 5C). At 3 months (SL 26 mm), the endochondral bones were developed into a mature skeleton in WT, however, in *fgfr3* mutants there were still some unossified cartilage such as ceratohyal and basihyal cartilage (Figure 5D). These data indicate that* fgfr3* is required for the patterning and shaping of pharyngeal arches.

**Figure 6:**
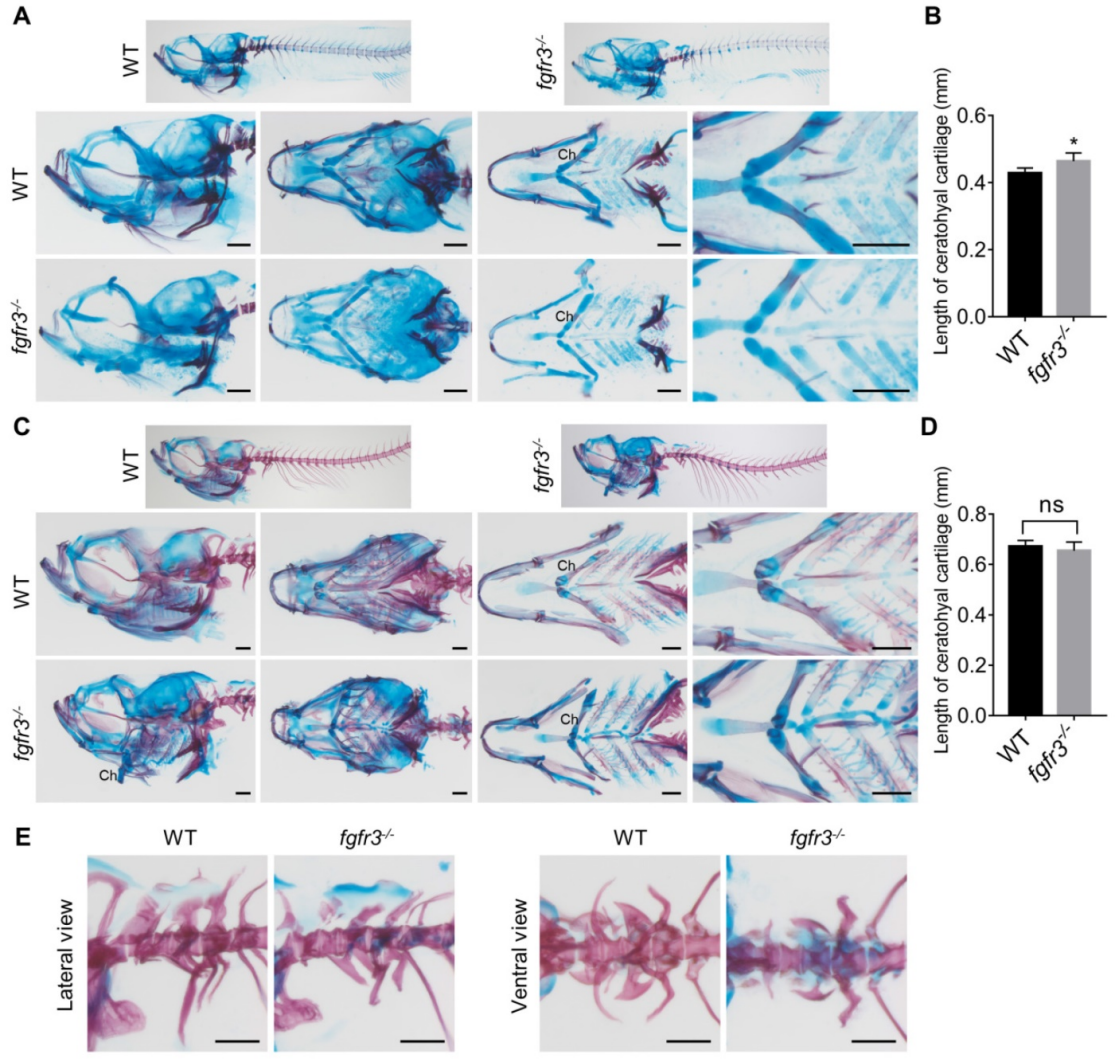
** The phenotype of *fgfr3* mutant zebrafish detected by Alizarin red and Alcian blue whole skeleton staining. (A, C)** Alizarin red and Alcian blue whole skeleton staining of the WT and *fgfr3* mutant at 20 dpf (SL 7.5 mm) (A) and 30 dpf (SL 10.0 mm) (C). Left two panels show the lateral view and dorsal view of the craniofacial bone. Right two panels show the dissected pharyngeal arches and the magnified ceratohyal bone. **(B, D)** Quantification of ceratohyal cartilage length for WT and* fgfr3* mutants at 20 dpf (SL 7.5 mm) (B) and 30 dpf (SL 10.0 mm) (D). *n* = 6, *p<0.05, no significance (ns). (E) Lateral view (left) and ventral view (right) of the Weberian apparatus of the WT and *fgfr3* mutant at 30 dpf (SL 10.0 mm). Ch: ceratohyal cartilage. Scale bar, 200 µm in A, C and E.

We also used Alizarin red and Alcian blue whole skeleton staining to further examine the development of craniofacial and axial skeleton in *fgfr3* mutants at 20 dpf (SL 7.5 mm) and 30 dpf (SL 10.0 mm) (Figure 6A, C). We found the similar skeleton changes in mutants as those observed using *in vivo* imaging. These data revealed that both the intramembranous ossification and endochondral ossification were inhibited in *fgfr3* mutants. Among them, the ossification of intramembranous bone in opercle, branchiostegal rays and dentary were strongly inhibited, while the endochondral ossification of ceratohyal, mentomeckelian, retroarticular, basihyal, hypobranchial, palatoquadrate and ceratobranchial bone were all delayed with abnormal morphology of mandibular arch and hyoid arch (Figure 6A, C). Interestingly, we found that the length of ceratohyal cartilage was increased in* fgfr3* mutants in contrast to WT at 20 dpf (SL 7.5 mm) (Figure 6B, D). The increased length of ceratohyal cartilage in mutant zebrafish is consistent with the increased long bone length in CATSHL syndrome patients and *Fgfr3*-deficient mice.

### *Fgfr3* mutation leads to abnormal hypertrophy and disarrangement of chondrocytes

**Figure 7:**
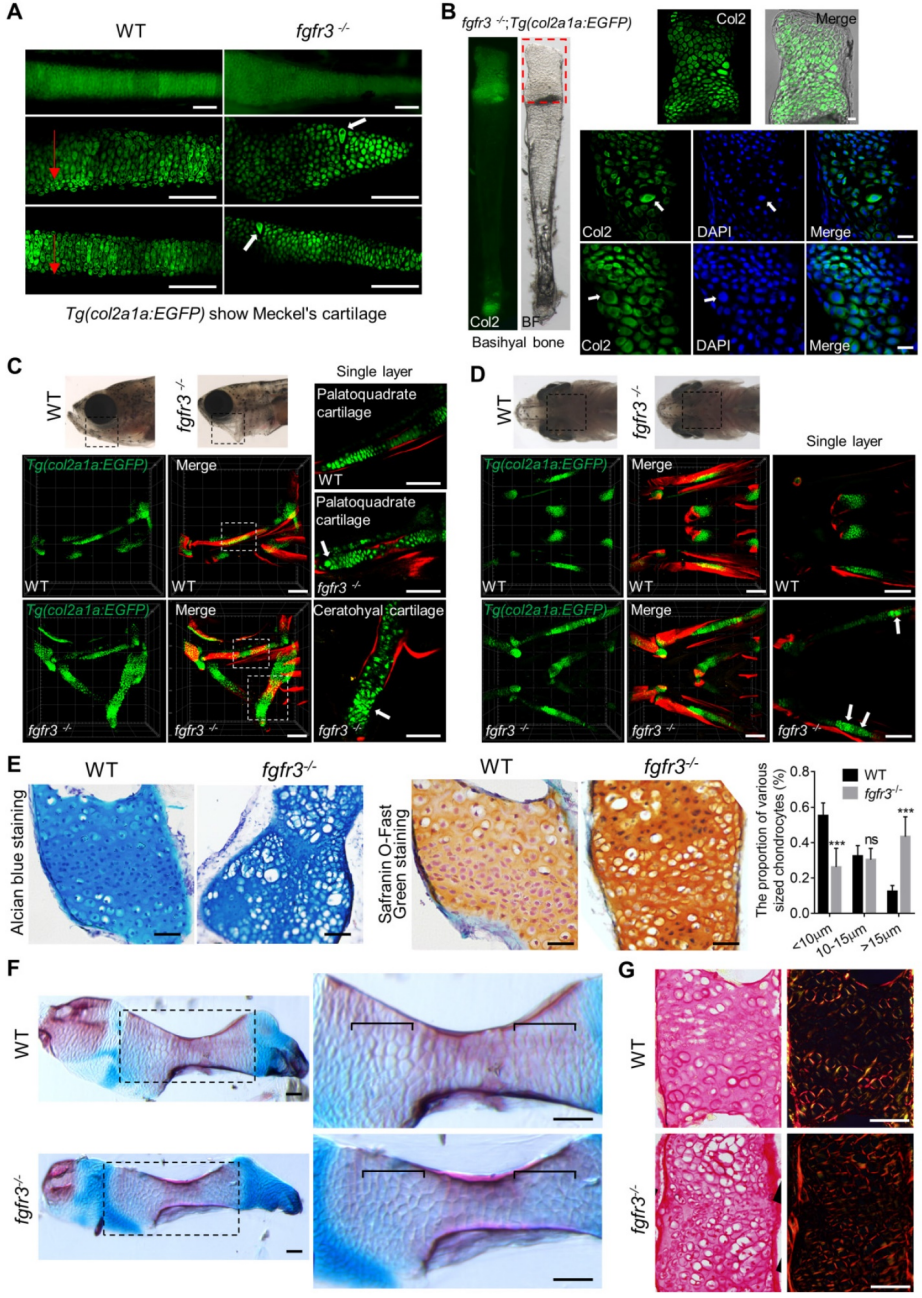
***Fgfr3* mutation leads to abnormal chondrocyte hypertrophy and arrangement. (A)** Images show the dissected Meckel's cartilage of WT and* fgfr3* mutants at 2 months (SL 18.0 mm) in *Tg(col2a1a:EGFP)* background. The top panel is the stereo fluorescence microscope image, the middle and bottom panel are the confocal images. Red arrows indicate the uniform arrangement of chondrocytes in WT and the white arrows indicate abnormal hypertrophy and disorganized chondrocyte orientation in *fgfr3* mutants. **(B)** Images show the dissected basihyal bone of *fgfr3* mutants at 2 months (SL 18.0 mm) in *Tg(col2a1a:EGFP)* background. The left panel shows the stereo fluorescence microscope image, the right panel shows the confocal images. White arrows indicate the abnormally enlarged chondrocytes and the nuclei in basihyal cartilage of *fgfr3* mutants. **(C-D)** Confocal imaging of WT and* fgfr3* mutants in *Tg(col2a1a:EGFP)* background live stained with Alizarin red at 30 dpf (SL 10.0 mm) in lateral view (C) and ventral view (D). Boxed regions in the top bright field images are magnified in the bottom 3D confocal image. The right panel show the single layer image. White arrows indicate abnormal hypertrophy and disarrangement of ceratohyal and palatoquadrate chondrocytes in *fgfr3* mutants. **(E)** Alcian blue staining (left) and Safranin O-Fast Green staining (right) of epiphysial growth plate of ceratohyal cartilage in WT and *fgfr3* mutants at 1.5 month (SL 14.0 mm). The right panel are the quantification of the proportion of various sized chondrocytes in epiphysial growth plates of ceratohyal cartilage. *n* = 5, ***p<0.001, no significance (ns). **(F)** Alizarin red and Alcian blue whole skeleton staining of dissected ceratohyal bone of WT and* fgfr3* mutants at 1.5 months (SL 14.0 mm). Boxed regions are magnified in the right panel. Brackets indicate that the chondrocytes in growth plate were ordered in WT and disorganized in *fgfr3* mutants. **(G)** Picric-sirius red staining for epiphysial growth plate of WT and* fgfr3* mutants, the left panel show the image from ordinary light microscope and the right panel show the image from polarized light microscopy. Scale bars: 100 µm in A, C and D, 20 µm in B, 50 µm in E-G.

To clarify the underlying mechanisms for the morphological abnormality of craniofacial skeleton in *fgfr3* mutants, we dissected and examined multiple craniofacial bones. We found that there was irregular chondrocyte orientation with resultant disorganized chondrocyte column in Meckel's and basihyal cartilage in *fgfr3* mutants, and some chondrocytes in these cartilage were abnormally hypertrophied (Figure 7A, B). In particular, the chondrocytes and their nuclei had variable sizes and shapes, some nuclei were abnormally enlarged in basihyal cartilage of *fgfr3* mutants (Figure 7B). Abnormal enlarged hypertrophic chondrocytes were also found in the palatoquadrate cartilage of 1-month mutants (SL 10.0 mm) (Figure 7C, D). Alcian blue and Safranin O-Fast Green staining revealed orderly arranged reserve, proliferation and hypertrophic chondrocyte zone in WT ceratohyal cartilage, which was completely disrupted in mutant cartilage with variably sized and shaped chondrocytes (Figure 7E). We also found that the proportion of chondrocytes with abnormally enlarged volume and hypertrophy was increased significantly in *fgfr3* mutants (Figure 7E). The disorganized epiphysial growth plates and chondrocytes were also confirmed by Alizarin red and Alcian blue whole skeleton staining in mutant zebrafish (Figure 7F).

To determine whether the organization of collagen fibers in cartilage was related with the disrupted chondrocyte arrangement in *fgfr3* mutant cartilage, we did Picric-sirius red staining for epiphysial growth plate and utilized polarized light microscopy, a common method to study collagen networks in tissues 42. We found that the chondrocytes in WT were well defined with symmetric and well-organized sharp fibers, however, the collagen fibers lost its symmetry with disrupted organization in *fgfr3* mutant chondrocytes (Figure 7G). The results suggest that *fgfr3* mutation affected the organization of collagen fibers in the cartilage matrix.

Toydemir et al. reported that there are osteochondroma in the long bones of several members of a family with CATSHL syndrome, and our previous study has found that loss of Fgfr3 function leads to the formation of multiple chondroma-like lesions in mice 43. We performed histological analyses on the epiphysial growth plate of ceratohyal cartilage at adult stage. Consistently, chondroma-like lesions such as cartilaginous lobules with irregular architecture and cellular pleomorphism composed of many small chondrocytes and large hypertrophic chondrocytes were also found in some* fgfr3* mutant zebrafish (Figure S6A). We did histological analyses of the joint between the precaudal vertebrae and the ribs, which consist of cartilaginous element and are formed through endochondral ossification. In contrast to the WT, some *fgfr3* mutants also showed chondroma-like lesions in the rib attached to the precaudal vertebrae (Figure S6B). Briefly, our results suggest that Fgfr3 similarly regulates chondrocyte differentiation and growth plate development in zebrafish as it does in mice and humans.

### *Fgfr3* knockout leads to abnormal development of swim bladder and auditory sensory organs

Swim bladder occupies the same position as the lungs of higher vertebrates and is regarded as homologous to the lungs of mammal 44. It has been reported that FGFR3 and FGFR4 function cooperatively to promote the formation of alveoli during lung development in murine 45,46. Interestingly, we found that the dysregulated development of swim bladder in *fgfr3* mutants. The swim bladder in the WT consist of an anterior and a posterior sac-like chambers, but in *fgfr3* mutants, there was only single round chamber, or even no chamber at all (Figure 3C, E). These data suggest that *fgfr3* deficiency may affect the formation of swim bladder in zebrafish.

A hallmark phenotype of CATSHL syndrome patients is progressive sensorineural hearing loss 1-3. *Fgfr3* knockout mice also have sensorineural deafness 11. We detected whether *fgfr3* mutant zebrafish also have abnormalities in auditory sensory organs. The auditory sensory organ of zebrafish consists of the inner ear, Weberian apparatus and lateral lines 47. The gross morphology and size of otoliths in the inner ear of *fgfr3* mutants were normal as the WT at 5 dpf (SL 4.0 mm) and 10 dpf (SL 5.0 mm) (Figure S2A and S6A). Using transgenic line* Tg(col2a1a:EGFP)* that labels the otic vesicle 28, we found that the early development of the otic vesicle including its size and morphology appeared to be normal in *fgfr3* mutants at 10 dpf (SL 5.0 mm) (Figure S7A-B). However, the Weberian apparatus, which include scaphium, claustrum, intercalarium, tripus bones and other elements, were maldeveloped in *fgfr3* mutants. The ossification of these bones was delayed with abnormal morphology from 20 dpf (SL 7.5 mm) to 30 dpf (SL 10.0 mm) (Figure 6E). Besides, at adult stage, the Weberian apparatus still had abnormal morphology in contrast to the WT as evaluated by micro-CT, Alizarin red and Alcian blue whole skeleton staining (Figure 3G, Figure S1A). The lengths of both tripus and suspensorium bone were reduced in *fgfr3* mutants. Furthermore, the anterior chamber of swim bladder was tightly attached to the Weberian apparatus in the WT, but not in the *fgfr3* mutants (Figure 3E). The Weberian apparatus transfers acoustic signals from the swim bladders to the inner ear that will increase the auditory sensitivity at high frequencies 47. The maldevelopment of Weberian apparatus and swim bladder may be involved in the reduced hearing ability. Above results suggest that the deafness in CATSHL syndrome patients may be related to the abnormal development of auditory sensory organs.

### *Fgfr3* mutation inhibits osteoblast proliferation, promotes chondrocyte proliferation and enhances IHH signaling

**Figure 8:**
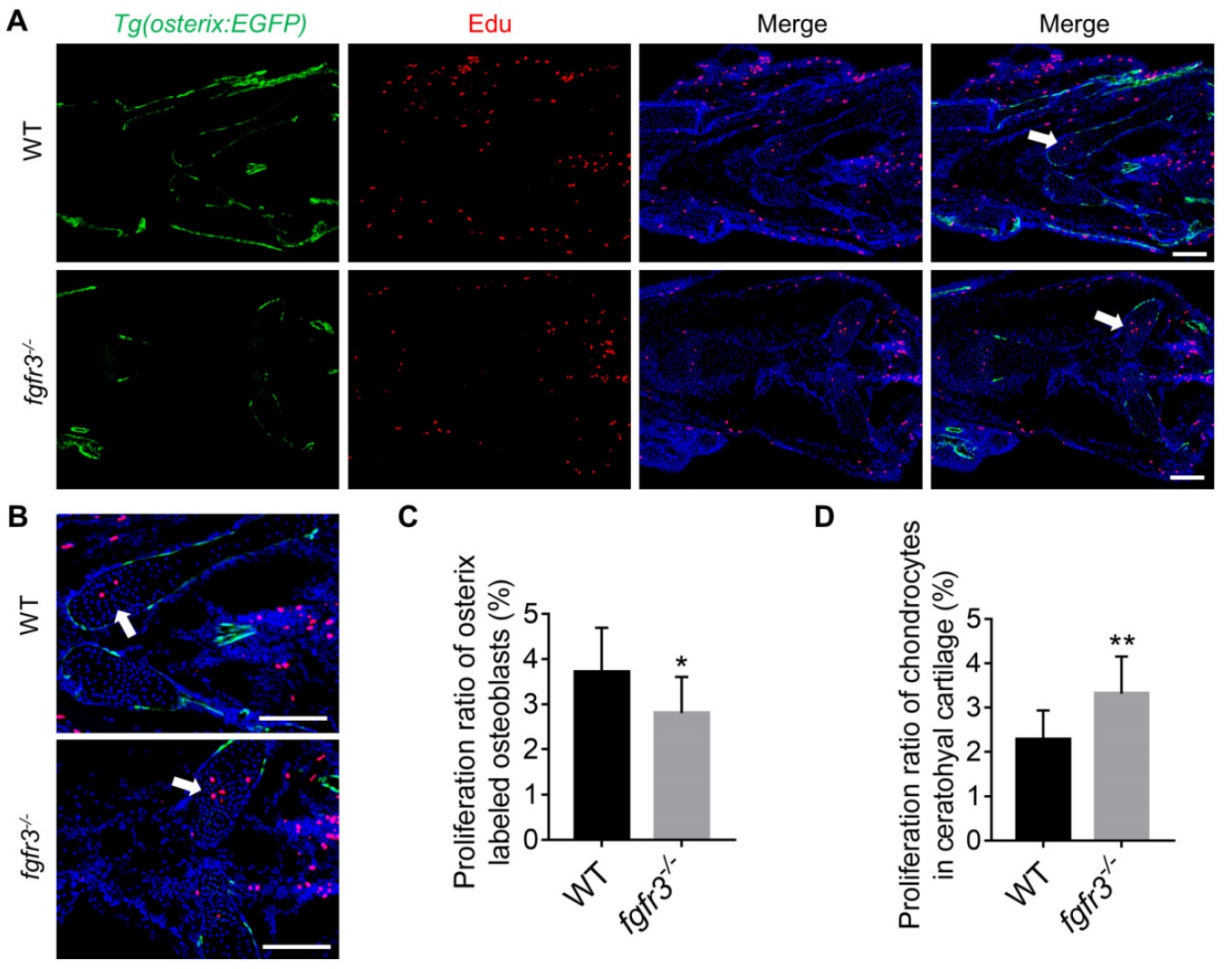
***Fgfr3* mutation promotes chondrocyte proliferation and inhibits osteoblast proliferation. (A)** Edu incorporation assay in WT and *fgfr3* mutants of *Tg(osterix:EGFP)* background at 1 month (SL 10.0 mm), **(B)** is the higher magnification of ceratohyal cartilage. White arrows indicate the increased proliferation of chondrocytes in ceratohyal cartilage of *fgfr3* mutants. **(C)** Quantification of the proliferation ratio of osterix labeled osteoblasts in (A), *n* = 10, *p < 0.05. **(D)** Quantification of the proliferation ratio of chondrocytes in ceratohyal cartilage in (A), *n* = 10, **p < 0.01. Scale bars: 100 µm in A.

Since intramembranous ossification and endochondral ossification were both inhibited in *fgfr3* mutants, we investigated the effects of *fgfr3* mutation on cell proliferation using Edu incorporation assay in *fgfr3* mutants of* Tg(osterix:EGFP)* transgenic background (Figure 8A). We found that the Edu labeled Osterix positive cells in the head region was decreased in 1-month-old (SL 10.0 mm) *fgfr3* mutants (Figure 8C), while the number of Edu labeled chondrocytes in the growth plate of ceratohyal cartilage was increased in *fgfr3* mutants in contrast to WT (Figure 8B, D). These data suggest that* fgfr3* mutation in zebrafish promotes chondrocyte proliferation but inhibits osteoblast proliferation.

**Figure 9:**
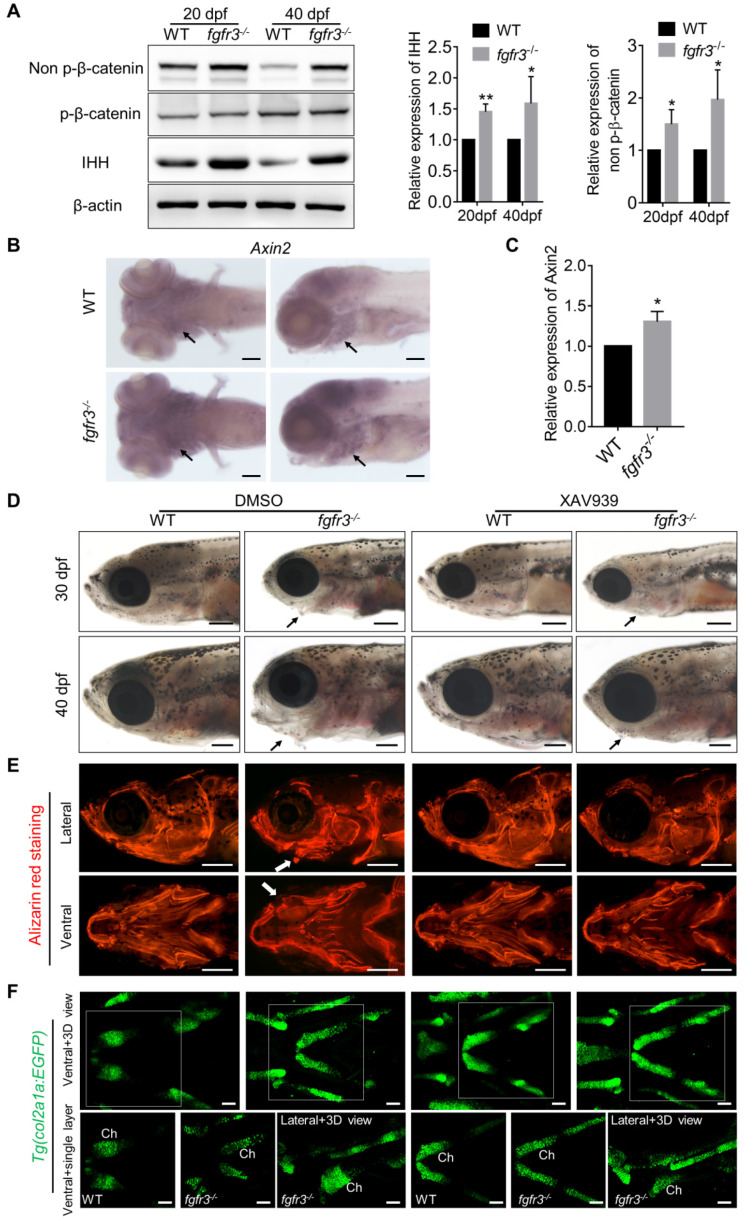
***Fgfr3* mutation upregulates canonical Wnt/β-catenin signaling and Wnt inhibition partially alleviates the phenotype of *fgfr3* mutants. (A)** Western blot detecting the protein levels of phosphorylated-β-catenin, non-phosphorylated β-catenin (activated β-catenin) and IHH in WT and mutant zebrafish at 20 dpf (SL 7.5 mm) and 40 dpf (SL 13.0 mm), β-actin was used as loading control. Quantitative analyses of the relative expressions of IHH and non-phosphorylated β-catenin are show in the right panel. *n* = 3 independent experiments, **p < 0.01, *p < 0.05. **(B)** Expression level of *axin2* examined by *in situ* hybridization at 7 dpf. Black arrows indicate pharyngeal cartilage. **(C)** RT-qPCR analysis of the expression level of *axin2* at 20 dpf (SL 7.5 mm). *n* = 3 independent experiments, *p < 0.05. **(D-F)** WT and *fgfr3* mutants treated with 2.5 µM XAV939 (right) or DMSO (left) from 10 dpf (SL 5.0 mm) to 20 dpf (SL 7.5 mm). (D) show the lateral view of head region detected by light microscopy at 30 dpf (SL 10.0 mm) (top) and 40 dpf (SL 13.0 mm) (bottom). Arrows indicate that the mandibular deformity with hyoid arch drooping toward the ventral side was partially rescued in XAV939-treated mutants. (E) show the lateral view (top) and ventral view (bottom) of craniofacial bone at 40 dpf (SL 13.0 mm) with living Alizarin red staining. (F) is the confocal images showing the ceratohyal cartilage (Ch) at 30 dpf (SL 10.0 mm) of WT and *fgfr3* mutants in *Tg (col2a1a:EGFP)* transgenic background. Boxed regions in the 3D confocal image are showed the single layer in the bottom. Scale bar, 100 µm in C, 400 µm in D and E, 50 µm in F.

IHH and PTHrP signaling are important regulators of the cartilage development 48. We and others have shown that constitutively activated FGFR3 results in downregulation of Ihh mRNA in growth plate chondrocytes 10, 49, 50. Moreover, our previous study in mice showed that FGFR3 deficiency causes multiple chondroma-like lesions by upregulating hedgehog signaling 43. Therefore, we examined whether IHH signaling is perturbed after *fgfr3* mutation in zebrafish. Western blot using the extracts from the head skeleton at 20 dpf (SL 7.5 mm) and 40 dpf (SL 13.0 mm) showed that Ihh protein was significantly increased in *fgfr3* mutants (Figure 9A). These results suggest that the enhanced Ihh signaling may be associated with the abnormal development of pharyngeal cartilage in *fgfr3* mutants.

### *Fgfr3* mutation upregulates canonical Wnt/β-catenin signaling and Wnt inhibition partially alleviates the phenotypes of *fgfr3* mutants

We next explored for the possible signaling pathway involved in skeletal maldevelopment of *fgfr3* mutants. Recent study has showed the functional link between Hh signaling and Wnt/β-Catenin signaling in skeletal growth and cartilage/bone tumor formation 51. Shung et al. found that gain-of-function mutations of FGFR3^K650E^ led to down-regulated of β-catenin level and activity in growth plate chondrocytes 52. Thus, we examined the levels of accumulated β-catenin protein in mutant and WT cartilage. Western blot using the extracts from the head skeleton at 20 dpf (SL 7.5 mm) and 40 dpf (SL 13.0 mm) showed that the level of non-phosphorylated β-catenin (activated-β-catenin) was remarkably up-regulated in *fgfr3* mutants, while phosphorylated-β-catenin had no significant change (Figure 9A). Furthermore, *in situ* hybridization showed that the level of Wnt/β‐catenin target gene *axin2* was increased in the pharyngeal cartilage of *fgfr3* mutants (Figure 9B). Consistently, RT‐qPCR analysis showed that the expression level of *axin2* was increased in head skeleton of *fgfr3* mutants at 20 dpf (SL 7.5 mm) (Figure 9C). These results suggest that Wnt/β-catenin signaling was up-regulated in *fgfr3* mutants.

We then asked whether pharmacological inhibition of Wnt/β-catenin signaling could rescue the craniofacial bone malformation caused by *fgfr3* mutation. We used a β-catenin inhibitor XAV939, which stimulates β-catenin degradation by stabilizing axin, one suppressor of β-catenin destruction complex 53,54. 2.5 µM XAV939 or DMSO control were used from 10 dpf (SL 5.0 mm) to 20 dpf (SL 7.5 mm). We analyzed their effect at 30 dpf (SL 10.0 mm) and 40 dpf (SL 13.0 mm), and found that the abnormal drooping of hyoid arch in *fgfr3* mutants was partially alleviated by XAV939 (Figure 9D and Figure S8A), the domed-shaped skulls and microcephaly also were partially alleviated (Figure 9D). Alizarin red staining showed that the asymmetrical morphology of pharyngeal arches in *fgfr3* mutants was partially corrected after XAV939 treatment (Figure 9E), but the delayed intramembranous ossification of opercle and branchiostegal rays was not significantly changed in XAV939-treated mutants (Figure 9E). We detected the distance between Meckel's and ceratohyal bone (M-Ch distance) and the angle between vertical and horizontal axis (V-H angle) of skulls after XAV939 treatment, and showed that XAV939 treatment partially rescued the mandibular asymmetry in *fgfr3* mutants (Figure S8B, C). We then used transgenic line* Tg(col2a1a:EGFP)* to detect the cartilage development, and revealed that XAV939-treated mutants had their chondrogenic dysplasia especially the dysplasia of ceratohyal cartilage partially alleviated. The abnormal arrangement and hypertrophy of chondrocytes in ceratohyal cartilage were both alleviated in XAV939-treated mutants (Figure 9F). These results suggest that skeletal dysplasia especially the chondrodysplasia in *fgfr3* mutants is associated with up-regulated Wnt/β-catenin signaling, which could be partially alleviated by inhibiting Wnt/β-catenin signaling.

## Discussion

The critical roles of FGFs/FGFR3 in human skeleton development and diseases have been well documented 4. It's established that FGFR3 negatively regulates the development of skeleton formed through endochondral ossification 6, 8. However, the underlying mechanism of the negative regulatory role of FGFR3 in endochondral bone development is not fully clarified, for example there is still dispute about the effect of FGFR3 on chondrocyte hypertrophy. Moreover, it is still highly debatable about the roles of FGFR3 in the development of skeleton formed through intramembranous ossification, i.e., skull vaults. For example, both mice with either FGFR3 deficiency or gain-of-function mutations have decreased bone mass and mineralization 10,13,55. Activated mutations of FGFR3 lead to craniosynostoses with macrocephaly and midfacial hypoplasia in patients and mouse models due to premature closure of cranial sutures and cranial base synchondrosis 36,37,56,57. While CATSHL syndrome patients are also reported to have microcephaly and wormian skull bones 2,3. In contrast, the *Fgfr3* deficient mice have no apparent phenotype in craniofacial skeleton 12,13. One important reason for these seemingly controversial phenotypes is that the mouse models and patients are not suitable for dynamically studying the pathological process especially the early maldevelopment of skeleton.

Because of its *in vitro* and fast development, and transparency, zebrafish is very good for dynamically studying the early development of skeleton 14,16,18. Moreover, the development of craniofacial skeleton has proven to be highly conserved from zebrafish to humans 22,23. These facts prompted us to utilize zebrafish to answer those yet to be clarified questions mentioned above.

In this study, we generated a zebrafish model mimicking human CATSHL syndrome by deleting *fgfr3* in zebrafish, to study the pathogenesis and potential therapeutic targets for CATSHL syndrome. We found that the *fgfr3* mutant zebrafish have severe craniofacial bone malformation, as evidenced by microcephaly with smaller cranial skull bones and delayed closure of cranial sutures, drooped hyoid arch and midface hypoplasia, which to some extent resembles the clinical manifestations of CATSHL syndrome patients 3. These changes suggest that, compared with mouse CATSHL model, our *fgfr3* knockout zebrafish seems to better mimic the craniofacial skeleton phenotypes of CATSHL syndrome.

Using this zebrafish model, we analyzed the early development process and clarified the underlying mechanisms of craniofacial bone malformation in *fgfr3* mutants. We first dynamically examined bone mineralization and osteoblast differentiation using transgenic fish labeling osteoblasts and live Alizarin red staining from 5 dpf (SL 4.0 mm) to 2 months (SL 18.0 mm). We found that the osteoblastic differentiation and mineralization is severely affected in *fgfr3* mutants. The intramembranous bones including opercle, branchiostegal rays, parietal and frontal bones were consisted of several small and separately located extra bones, which were unable to develop into fully matured single bone in *fgfr3* mutants. The proliferation of osteoblasts and expressions of genes related to osteoblast differentiation were down-regulated, which may be responsible for the severely delayed ossification of intramembranous bones in* fgfr3* mutants. In particular, the skull vault in *fgfr3* mutants consisted of multiple, irregular cranial bones, which is very similar to that of *osterix/sp7* mutants with extra sutures and irregular skull bones 58, suggesting that the skull malformations in *fgfr3* mutants may be related to the delayed osteoblast differentiation and bone maturation due to down-regulated osterix expression. Besides, the delayed scale formation in *fgfr3* mutants also confirms that *fgfr3* mutation inhibits osteoblast differentiation and bone mineralization in zebrafish.

**Figure 10:**
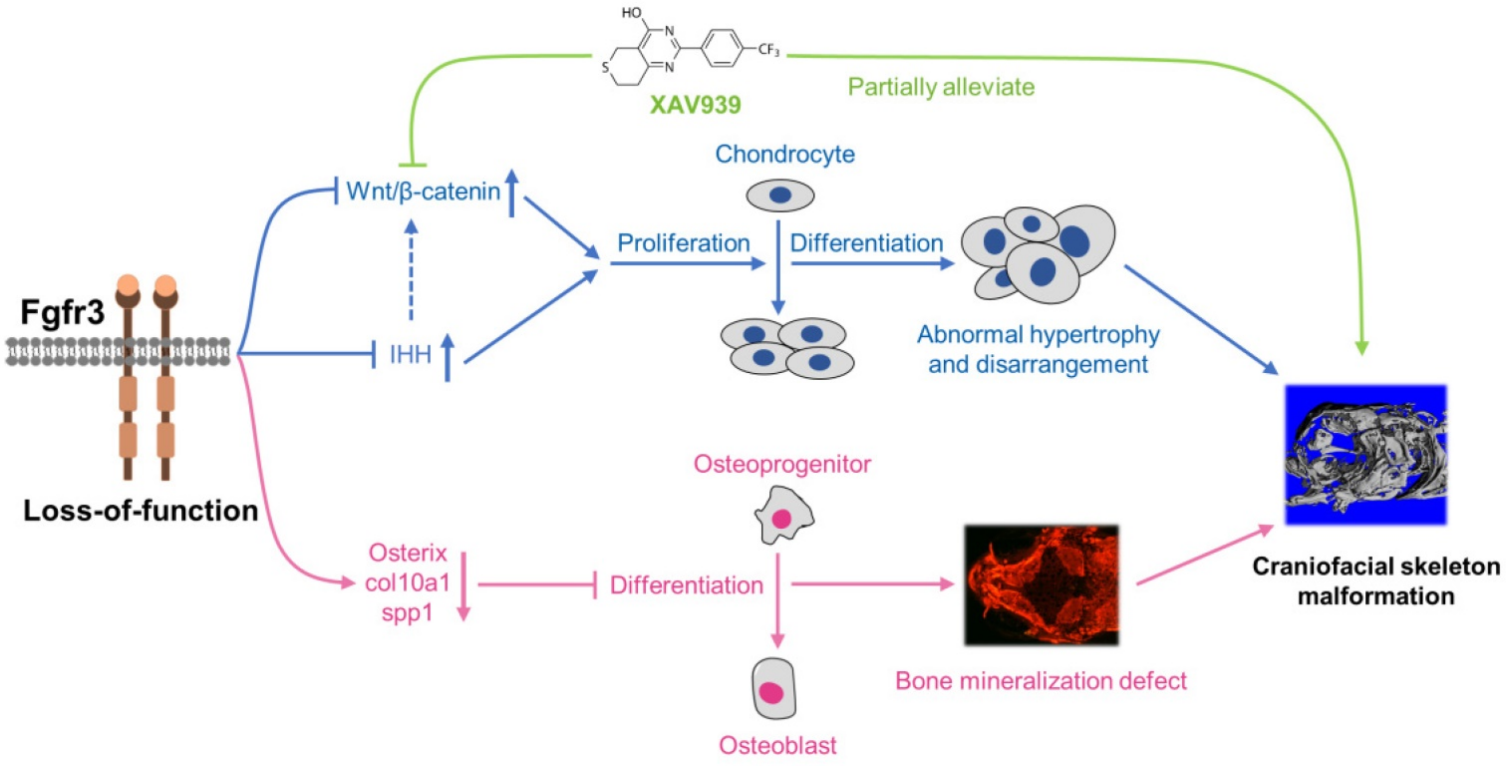
** Schematic diagram of the mechanisms underlying the role of *fgfr3* in zebrafish skeleton development.** Deletion of *Fgfr3* in zebrafish results in enhanced IHH signaling and up-regulated canonical Wnt/β-catenin signaling that may lead to increased chondrocyte proliferation, abnormal hypertrophy and disordered arrangement of chondrocytes in growth plates. *Fgfr3* mutation leads to decreased proliferation and differentiation of osteoblasts and decreased mineralization in skull bone. A combination of above mechanisms may lead to disrupted chondrogenesis and bone ossification resulting in craniofacial skeleton malformation in *fgfr3* mutant zebrafish.

In addition to the role of osteogenesis, the abnormal chondrogenesis of jaw bone and skull base cartilage is also involved in the craniofacial malformation 34. We then dynamically examined the early development of cartilage from 5 dpf (SL 4.0 mm) to 1 month (SL 10.0 mm) using *Tg(col2a1a:EGFP)* that label chondrocytes. We found that the ordered arrangement of chondrocytes was gradually disrupted with some abnormally hypertrophied chondrocytes in *fgfr3* mutants. The chondrocyte zone of ceratohyal cartilage growth plate was completely disrupted with chondrocytes of variable sizes and shapes. We suspect that the gradually maldeveloped cartilage structure may lead to mechanical changes of jaw cartilage, resulting in drooped hyoid arch and malformed jaw bone, which may also be related to the high arched palate in CATSHL syndrome patients 3. Besides, we also found delayed synchondrosis closure and irregular morphology in the cranial base of *fgfr3* mutants. In brief, we revealed disrupted chondrogenesis in the mutants, which, together with the dysregulated bone ossification of skull bones, results in the craniofacial skeleton malformation in *fgfr3* mutant zebrafish (Figure 10).

We explored the possible signaling pathway involved in the skeletal maldevelopment of *fgfr3* mutants. FGFR3 has close crosstalk with several signaling pathways that play critical roles in regulating skeleton development, including IHH, PTHLH/PTHrP, BMP and IGF1 4,59,60. Firstly, we found that IHH signaling was increased in mutant zebrafish. As recent study showed that Wnt/β-catenin signaling acts downstream of Hh signaling in skeletal growth and tumor formation 51, we thus speculated that *fgfr3* deletion may also exert its effect on skeleton development through modulating Wnt/β-catenin signaling. Indeed, we found that canonical Wnt/β‐catenin signaling was up-regulated in *fgfr3* mutants, and pharmacological inhibition of Wnt/β‐catenin could partially correct the phenotype of craniofacial malformation in *fgfr3* mutants including the abnormal arrangement of chondrocytes, although the delayed intramembranous bone ossification was not significantly rescued. These data indicate that up-regulated Wnt/β-catenin signaling in *fgfr3* mutants may be mainly involved in the chondrodysplasia phenotype, while the disrupted bone ossification may be result from other mechanism. We found that XAV939 treatment also partially rescue the domed-shaped skulls and microcephaly of *fgfr3* mutants, we speculated that this is via improving the chondrogenic phenotype such as in jaw cartilage and cranial base cartilage, which may lead to improvement of cranial skeletal asymmetry and abnormal mechanics in *fgfr3* mutants. In addition, the rescue of drooped hyoid arch could improve the malformed mouth, which may also improve the overall skull phenotype by improving food intake in* fgfr3* mutants.

So far, the crosstalk between FGF/FGFR3 pathways and Wnt/β-catenin signaling in chondrogenesis is not fully understood. Shung et al. found that gain-of-function mutation of FGFR3^K650E^ led to down-regulation of β-catenin levels and activity in growth plate chondrocytes, as well as decreased β-catenin levels and transcriptional activity in cultured mesenchymal cells 52. In contrast, some studies in cultured chondrocytes showed that FGF signaling activates Wnt/β-catenin signaling through MAP kinase-mediated phosphorylation of LRP6 61, 62. Since there are no studies dissecting the role of Wnt signaling in the skeleton maldevelopment resulting from FGFR3 deletion, our results for the first time reveal that *fgfr3* deficiency upregulates the Wnt/β-catenin pathway, which may be involved in the pathogenesis of the abnormal skeleton phenotype in CATSHL syndrome. However, the mechanism through which *fgfr3* mutation upregulates Wnt/β‐catenin signaling remains elusive, whether Fgfr3 negatively modulates Wnt pathway in human and mouse models requires additional studies.

The dwarfism phenotypes in ACH patients and mouse models, as well as the skeletal overgrowth with tall stature in CATSHL syndrome patients and *Fgfr3* deficient mice strongly demonstrate that FGFR3 is a negative regulator of development of endochondral bone 4,8. However, the body size of *fgfr3* mutant fish was smaller at adult stage, and some *fgfr3* mutants even died before adult stage. These may be due to the eating disorders resulting from craniofacial bone maldevelopment especially the jaw bone malformation. In addition, FGFR3 mainly regulates growth plate development of the long bones and vertebral bodies, while the zebrafish have no limbs and long bones, and the formation of their vertebral bodies is not through endochondral ossification as zebrafish vertebral body has no growth plate. All these differences between zebrafish and mammals may be responsible for the absence and presence of longer body length in *fgfr3* mutant zebrafish and mice/humans. However, it is important that some craniofacial bones in zebrafish such as the ceratohyal bones have growth plates and are formed through endochondral ossification like humans 20. *Fgfr3* expression was also detected in the proliferative and hypertrophic chondrocytes of growth plates of these zebrafish bones 27. Consistently, we found that the length of ceratohyal bone in *fgfr3* mutants was significantly increased with enhanced proliferation of its growth plate chondrocytes in contrast to WT at 20 dpf (SL 7.5 mm). Our data suggest that the development of cartilage is conserved between zebrafish and mammals, and our* fgfr3* deficient zebrafish model can be used to study the role of FGFR3 in chondrogenesis and screen therapeutic molecules for FGFR3 related genetic diseases including CATSHL and ACH by taking the advantages of zebrafish.

Similar to the variation in the phenotype severity in CATSHL syndrome patients 3,* fgfr3* mutant zebrafish also exhibited varying degrees of craniofacial bone malformations. The reasons for the variable phenotypes in mutant zebrafish are not clear presently. We think one important reason is the secondary effects of malformed mouth, which will strongly affect the food intaking. Indeed, we noticed that the body length of WT and *fgfr3* mutant had no significant change at 1 month (SL 10.0 mm), hereafter, the cranial deformity and phenotypic differences gradually appeared apparently.

The zebrafish is recognized as an excellent model system for the study of the development of the vertebrate inner ear and its functions of balance and hearing. Many of the molecular mechanisms of inner ear development, endolymph generation, and hair cell development and function in zebrafish are conserved with those in the mammals 47. As hearing loss is a hallmark features of CATSHL syndrome patients, although we found that the gross morphology and size of otoliths and otic vesicle appeared to be normal in *fgfr3* mutants, the Weberian apparatus and swim bladder were abnormally maldeveloped. These malformations may lead to reduced hearing function in *fgfr3* mutant zebrafish. Besides, the larval zebrafish jaw is evolutionarily homologous to the mammalian middle ear ossicles 63. The disrupted patterning and shaping of pharyngeal arches in *fgfr3* mutants indicate that FGFR3 may regulate the development of mammalian middle ear ossicles. In *Fgfr3* knockout mice the inner ear defects include failure of pillar cell differentiation and tunnel of Corti formation that result in profound deafness 11. However, whether the hair cell have developmental defect in the inner ear of *fgfr3* mutant zebrafish needs more studies.

In conclusion, we generated a zebrafish model for CATSHL syndrome that partially resembles the clinical manifestations especially the craniofacial malformation of patients with CATSHL syndrome. Using this zebrafish model, we found that *fgfr3* deficiency upregulates canonical Wnt/β‐catenin signaling, and inhibition of Wnt/β‐catenin signaling could partially alleviate the phenotypes of *fgfr3* mutants. Our zebrafish model for CATSHL syndrome will help to better understand the underlying pathogenetic mechanisms of FGFR3 related human genetic syndromes including CATSHL and ACH, and explore their possible therapies.

## Supplementary Material

Supplementary figures.

## Abbreviations

ACH: achondroplasia
CATSHL syndrome: camptodactyly, tall stature and hearing loss syndrome
dpf: days post fertilization
FGFR3: fibroblast growth factor receptors 3
hpf: hours post fertilization
SL: standard length
WT: wild-type
