# Supplementary material for: Fgfr3 mutation disrupts chondrogenesis and bone ossification in zebrafish model mimicking CATSHL syndrome partially via enhanced Wnt/β-catenin signaling
Source: Theranostics. 2020 May 30;10(16):7111–30. doi: 10.7150/thno.45286 (PMC7330844; doi:10.7150/thno.45286)
Supplement: Supplementary file 1 — Supplementary figures. [file thnov10p7111s1.pdf]

Supplementary Information

Fgfr3 mutation disrupts chondrogenesis and bone ossification in zebrafish model mimicking CATSHL syndrome partially via enhanced Wnt/β-catenin signaling

Xianding Sun[1#], Ruobin Zhang[1#], Hangang Chen[1#], Xiaolan Du[1], Shuai Chen[1], Junlan Huang[1], Mi Liu[1], Meng Xu[1], Fengtao Luo[1], Min Jin[1], Nan Su[1], Huabing Qi[1], Jing Yang[1], Qiaoyan Tan[1], Dali Zhang[1], Zhenhong Ni[1], Sen Liang[1], Bin Zhang[1], Di Chen[2], Xin Zhang[3], Lingfei Luo[4*], Lin Chen[1*] and Yangli Xie[1*]

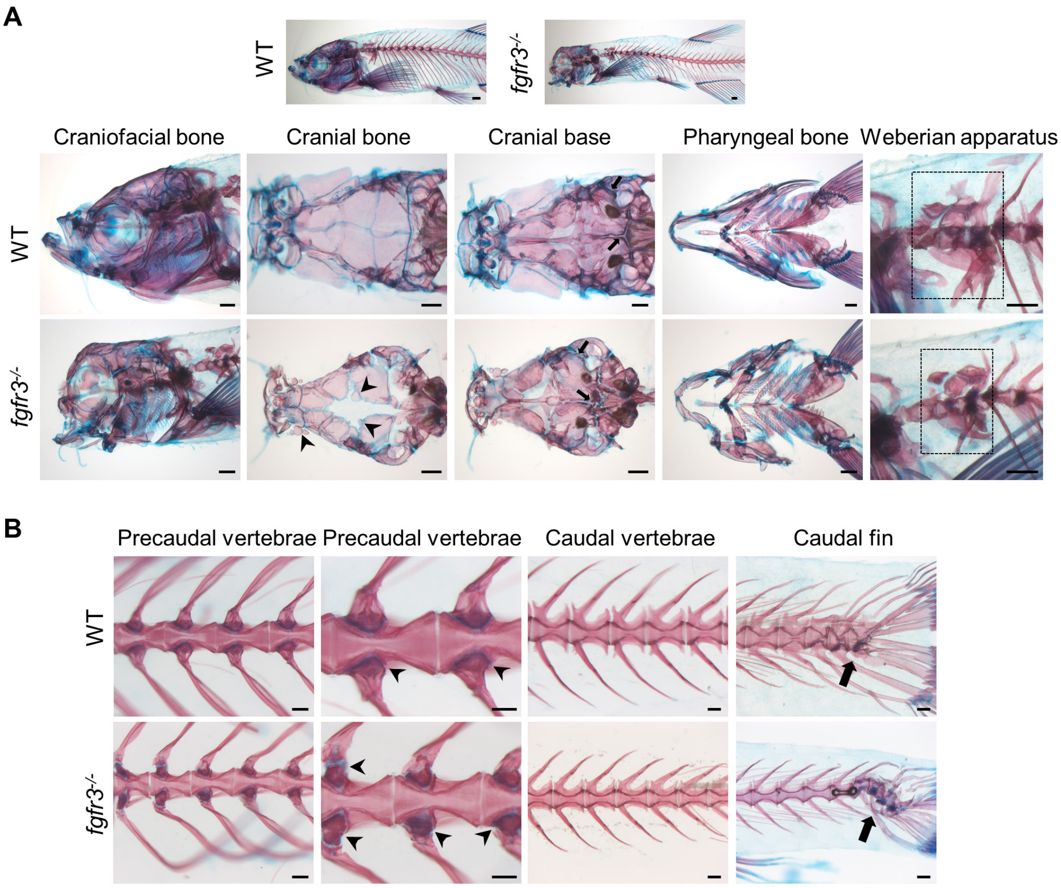

Figure S1. The phenotype of *fgfr3* mutant zebrafish detected by Alizarin red and Alcian blue whole skeleton staining. (A, B) Alizarin red and Alcian blue whole skeleton staining of the WT and *fgfr3* mutant. (A) showed the craniofacial bone, cranial bone, cranial base and pharyngeal bone, Weberian apparatus, respectively. Black arrowheads indicate some small bone islands at the margin of the cranial bone. Black arrows indicate the delayed synchondrosis closure and irregular morphology in *fgfr3* mutants. The dotted boxes indicate the Weberian apparatus. (B) showed the ventral view of precaudal vertebrae, magnified image of precaudal vertebrae, lateral view of caudal vertebrae and caudal fin, respectively. Black arrowheads indicate joint between the precaudal vertebrae and the ribs. Black arrows indicate the caudal fin vertebrae were slight bending with abnormal morphology. Scale bars: 500 μm in A and 200 μm in B.

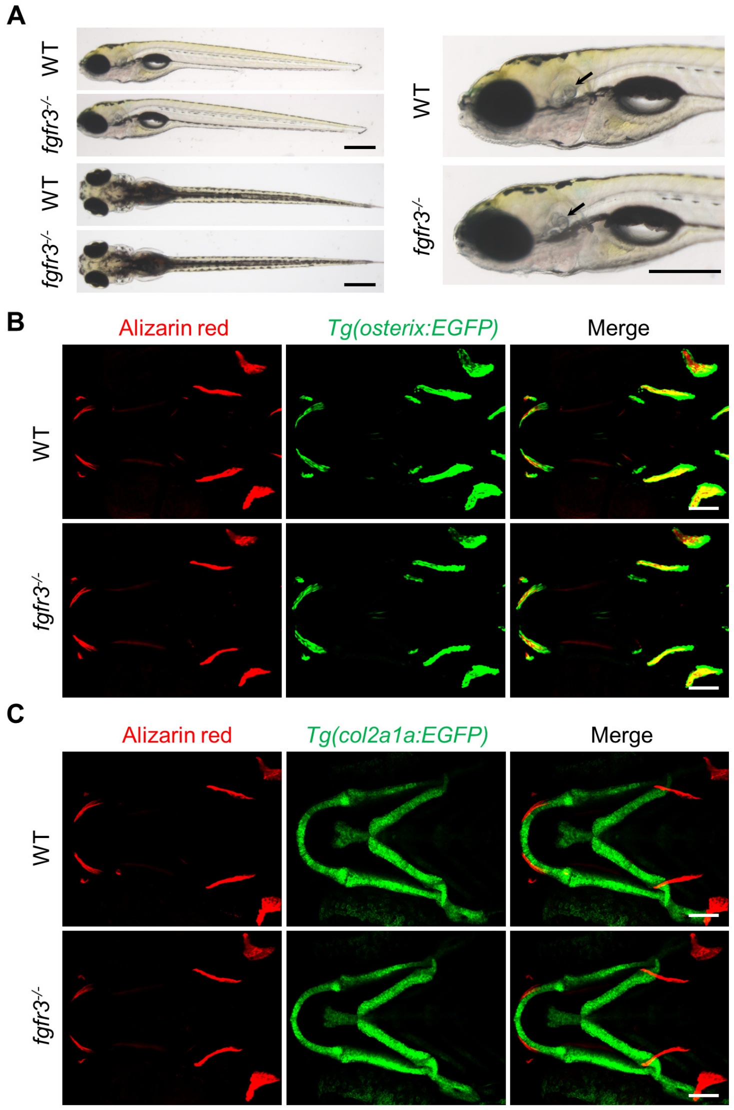

Figure S2. Bright field and transgenic fish showed no significant difference between WT and *fgfr3* mutants at 5 dpf. (A) Bright field images revealed no significant difference of gross appearance between WT and *fgfr3* mutants at 5 dpf. Black arrows indicate the otoliths. (B) Confocal imaging of WT and *fgfr3* mutants in *Tg(osterix:EGFP)* background live stained with Alizarin red at 5 dpf. (C) Confocal imaging of WT and *fgfr3* mutants in *Tg(col2a1a:EGFP)* background live stained with Alizarin red at 5 dpf. Scale bars: 400 μm in A, 100 μm in B and C.

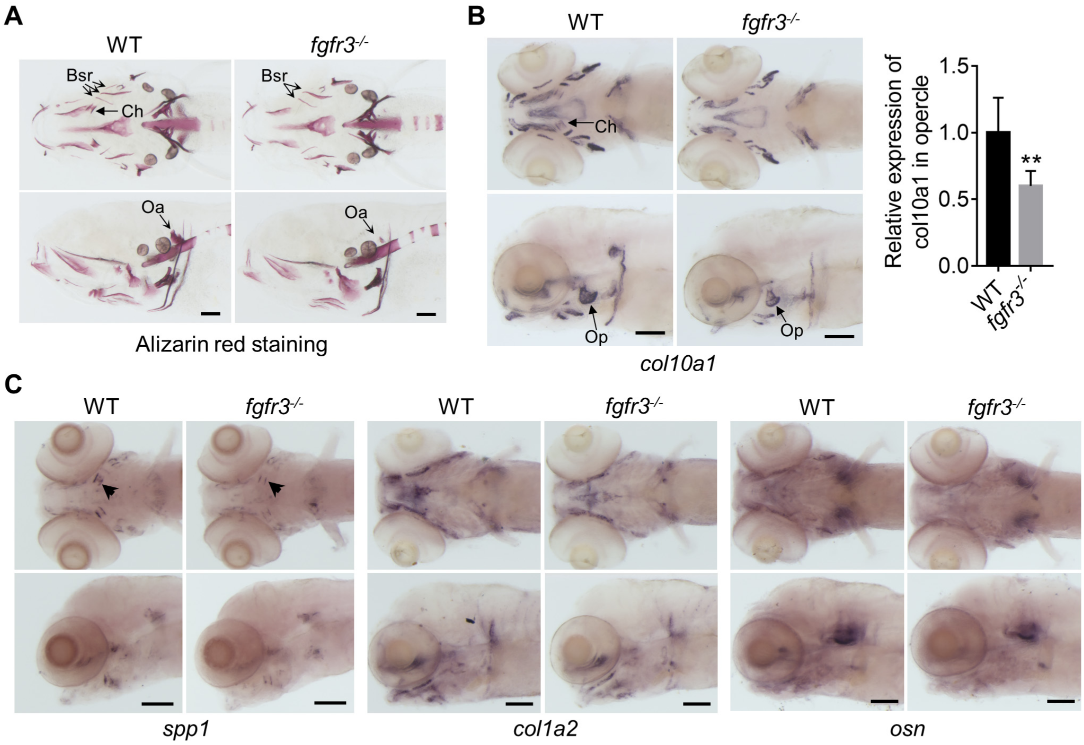

Figure S3. The phenotype of WT and *fgfr3* mutants detected by Alizarin red staining and *in situ* hybridization. (A) Alizarin red staining of WT and *fgfr3* mutants at 10 dpf. (B) *In situ* hybridization of *col10a1* in WT and *fgfr3* mutants at 8 dpf. The right panel are the quantification of relative expression of *col10a1* in opercle. $n = 8$, $^{**}p < 0.01$. (C) The left panel are the *in situ* hybridization of *osteopontin* (*spp1*) in WT and *fgfr3* mutants at 10 dpf. Note that the expression of *spp1* in ceratohyal bone (arrowheads) was decreased in *fgfr3* mutants in contrast to WT. The right two panels are the *in situ* hybridization of *col1a2* and *osteonectin* (*osn*) in WT and *fgfr3* mutants at 8 dpf. *Abbreviations*: Bsr, branchiostegal rays; Ch, ceratohyal bone; Oa, occipital arch; Op, opercle. Scale bars: 100 μm in A-C.

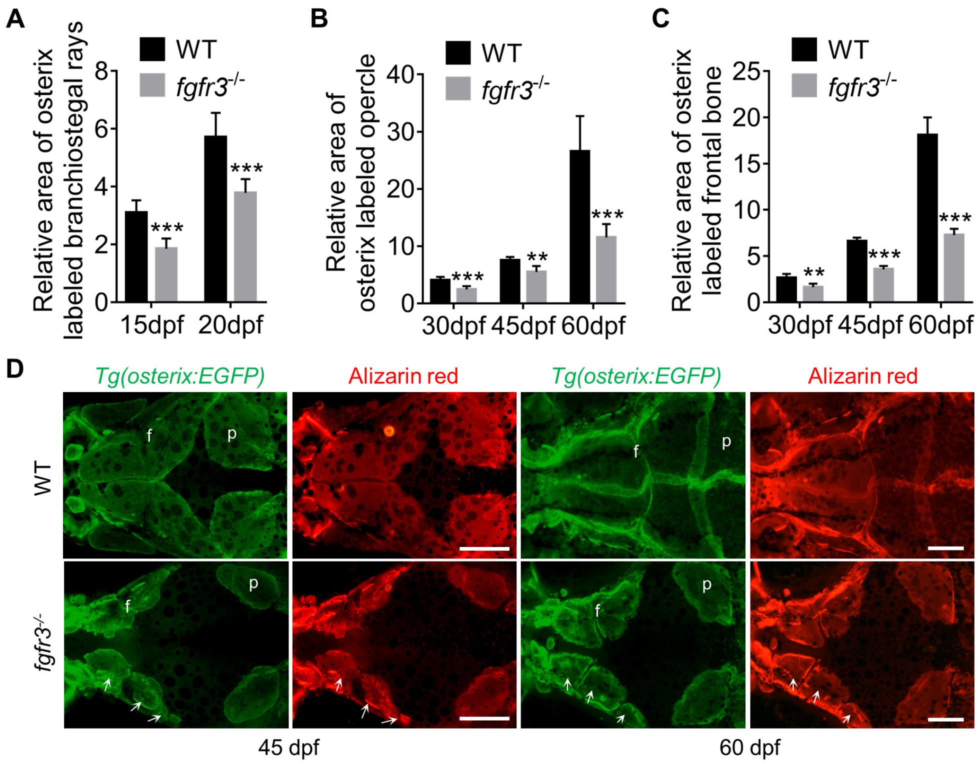

Figure S4. ***Fgfr3* is required for the timely bone ossification.** (A) Quantification of the relative area of osterix labeled branchiostegal rays for WT and *fgfr3* mutants at 15 dpf (SL 6.0 mm) and 20 dpf (SL 7.5 mm). (B) Quantification of the relative area of osterix labeled opercle for WT and *fgfr3* mutants at 30 dpf (SL 10.0 mm), 45 dpf (SL 14.0 mm) and 60 dpf (SL 18.0 mm). (C) Quantification of the relative area of osterix labeled frontal bone for WT and *fgfr3* mutants at 30 dpf (SL 10.0 mm), 45 dpf (SL 14.0 mm) and 60 dpf (SL 18.0 mm). $n = 6$ for A-C, $^{**}p < 0.01$, $^{***}p < 0.001$. (D) Magnified image of Figure 4E showed the parietal (p) and frontal (f) bones of WT and *fgfr3* mutants in dorsal view at 45 dpf (SL 14.0 mm) (left) and 60 dpf (SL 18.0 mm) (right). White arrows indicate mutants. Scale bars: 400 μm in D.

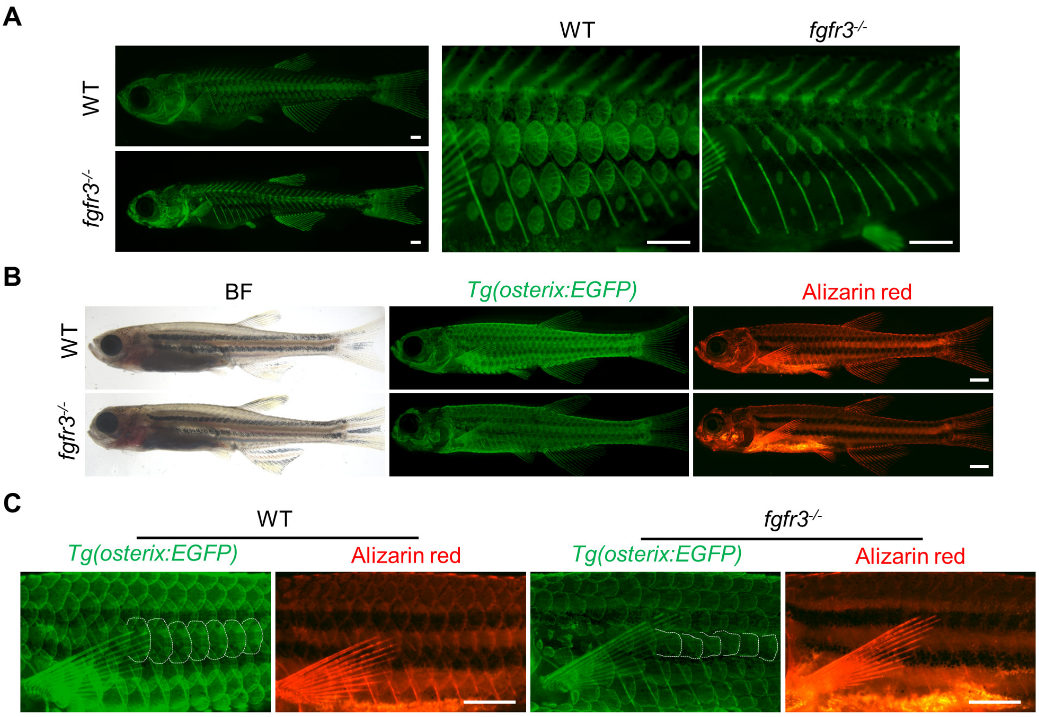

Figure S5. *Fgfr3* knockout inhibits scale formation. (A) Images showing the scale formation of WT and *fgfr3* mutants in *Tg(osterix:EGFP)* background at 35 dpf (SL 12.0 mm). (B, C) Stereo fluorescence microscope imaging of WT and *fgfr3* mutants in *Tg(osterix:EGFP)* background live stained with Alizarin red at 50 dpf (SL 16.0 mm). Higher magnification of the scale is in (C). The white dashed lines show the outline of the scale. Scale bar, 400 µm in A, 1 mm in B and C.

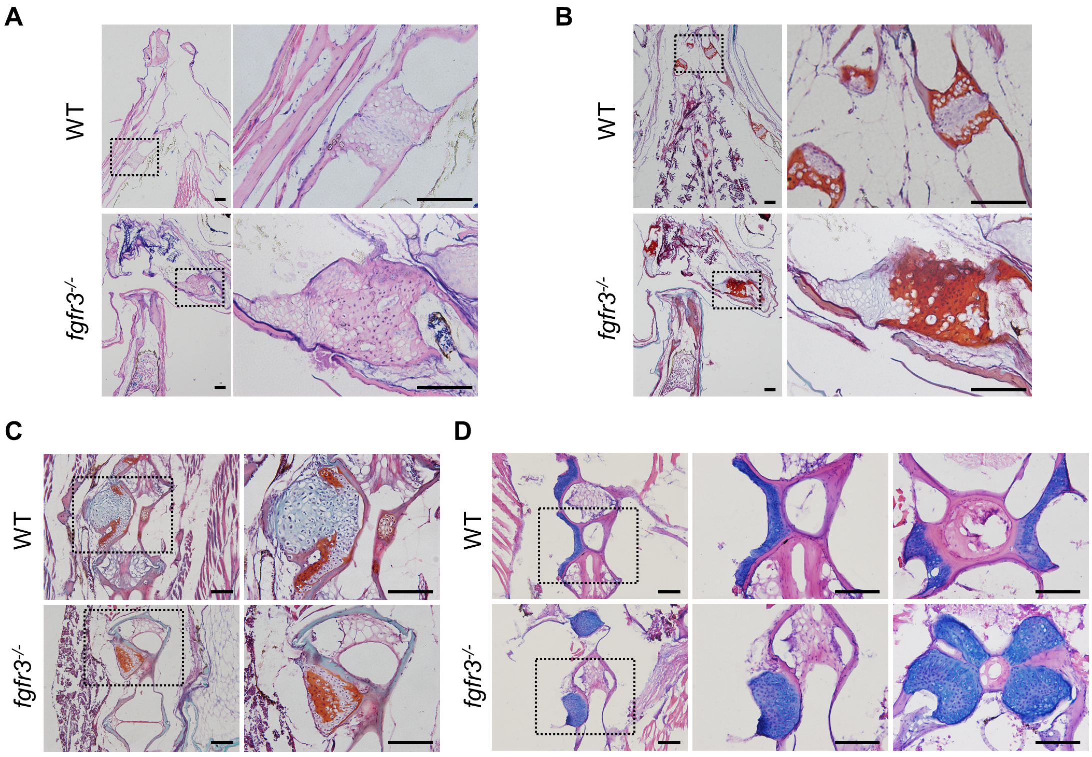

Figure S6. *Fgfr3* knockout in zebrafish leads to multiple chondroma-like lesions in ceratohyal cartilage and the joint between the precaudal vertebrae and the ribs. (A, B) Hematoxylin-Eosin (A) and Safranin O-Fast Green staining (B) for sagittal section of the epiphysial growth plate of ceratohyal cartilage at adult stage. Boxed regions are magnified in the right panel. (C) Safranin O-Fast Green staining for sagittal section of the joint between the precaudal vertebrae and the ribs. (D) Hematoxylin-Eosin staining for sagittal section or cross section of the joint between the precaudal vertebrae and the ribs. Boxed regions are magnified in the right panel. Scale bar, 200 μm in A and B, 100 μm in C and D.

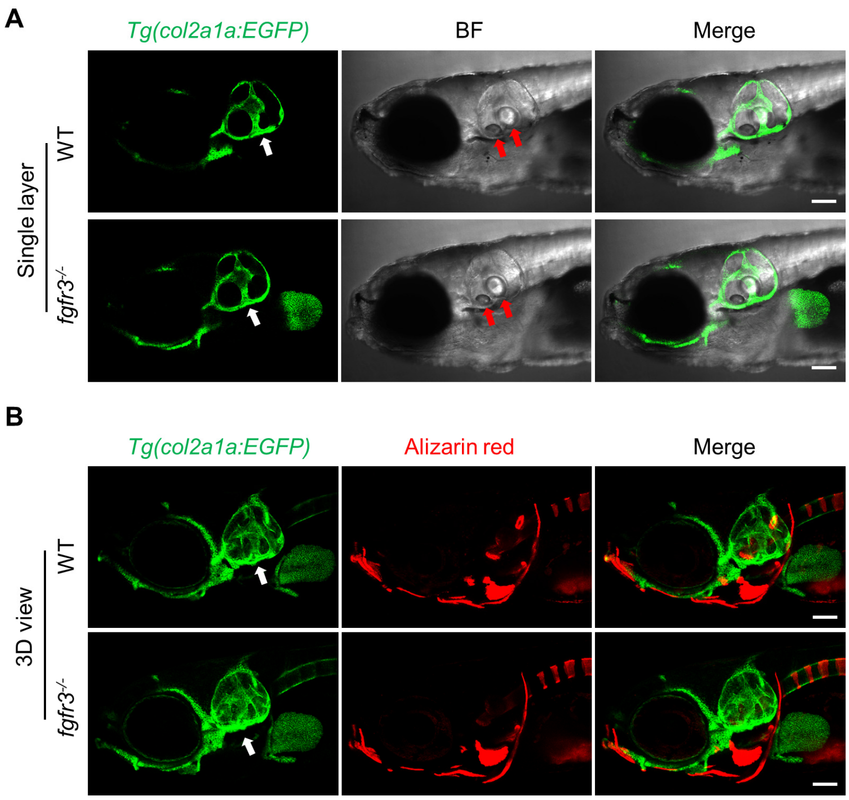

Figure S7. The phenotype of otic vesicle and otolith in WT and *fgfr3* mutants. (A, B) Confocal imaging of WT and *fgfr3* mutants in *Tg(col2a1a:EGFP)* background showed the otic vesicle (white arrow) and otolith (red arrow) in lateral view at 10 dpf. (A) showed the single layer and (B) showed the 3D view live stained with Alizarin red. The middle panel was the bright field (BF) images in (A). Scale bars: 100 µm in A and B.

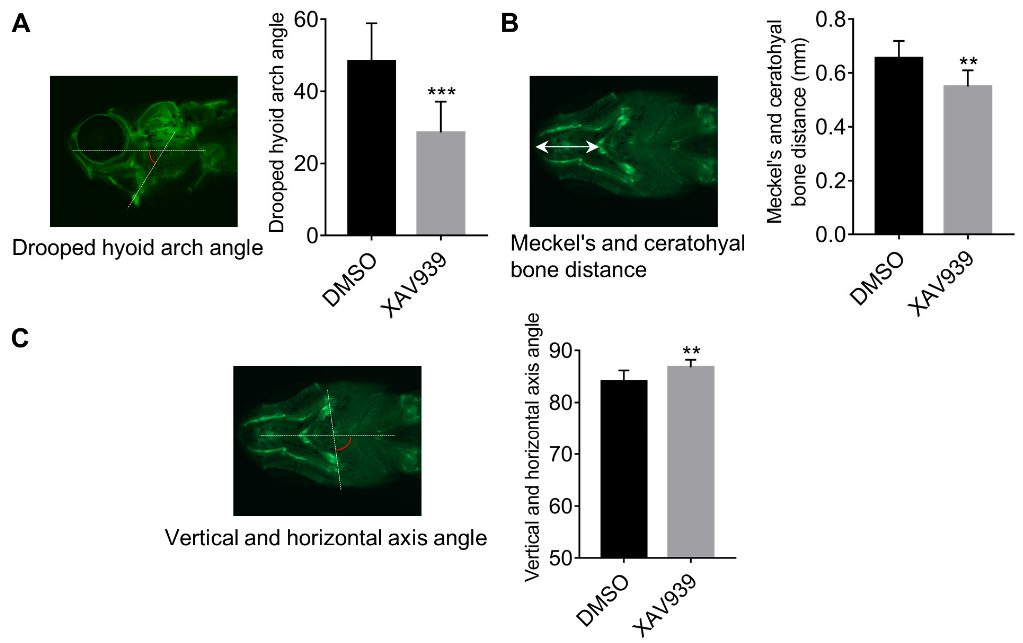

Figure S8. Wnt inhibition partially alleviates the phenotype in *fgfr3* mutants. (A) Quantification of drooped hyoid arch angle for *fgfr3* mutants at 40 dpf (SL 13.0 mm) after treatment with XAV939 or DMSO. (B) Quantification of distance between Meckel's and ceratohyal bone for *fgfr3* mutants at 40 dpf (SL 13.0 mm) after treatment with XAV939 or DMSO. (C) Quantification of angle between vertical and horizontal axis of skull for *fgfr3* mutants at 40 dpf (SL 13.0 mm) after treatment with XAV939 or DMSO. $n = 10$, **$p < 0.01$, ***$p < 0.001$.
